# Oncogenic GALNT5 confers FOLFIRINOX resistance via activating the MYH9/ NOTCH/ DDR axis in pancreatic ductal adenocarcinoma

**DOI:** 10.1038/s41419-024-07110-w

**Published:** 2024-10-21

**Authors:** Qinyuan Jia, Yuheng Zhu, Hongfei Yao, Yifan Yin, Zonghao Duan, Jiahao Zheng, Ding Ma, Minwei Yang, Jianyu Yang, Junfeng Zhang, Dejun Liu, Rong Hua, Yanmiao Huo, Xueliang Fu, Yongwei Sun, Wei Liu

**Affiliations:** 1 Department of Biliary-Pancreatic Surgery, Ren Ji Hospital, School of Medicine, Shanghai Jiao Tong University, Shanghai, 200127 P.R. China; 2 Department of Hepato-Biliary-Pancreatic Surgery, General Surgery, Huadong Hospital Affiliated to Fudan University, Shanghai, 200040 P.R. China; 3 State Key Laboratory of Systems Medicine for Cancer, Shanghai Cancer Institute, Ren Ji Hospital, School of Medicine, Shanghai Jiao Tong University, Shanghai, 200240 P.R. China

**Keywords:** Cancer therapeutic resistance, Tumour biomarkers

## Abstract

Chemotherapy resistance has been a great challenge in pancreatic ductal adenocarcinoma(PDAC) treatments. Current first-line chemotherapy regimens for PDAC include gemcitabine-based regimens such as AG regimen (albumin paclitaxel and gemcitabine), fluorouracil-based regiments such as FOLFIRINOX regimen ((5-fluorouracil5-FU), oxaliplatin, Irinotecan) and platinum-based regimens for patients with *BRCA* mutations. large amounts of work have been done on exploring the mechanism underlying resistance of gemcitabine-based and platinum-based regimens, while little research has been achieved on the mechanism of FOLFIRINOX regimens resistance. Hence, we identified Polypeptide N-Acetylgalactosaminyltransferase 5, (*GALNT5*) as a vital regulator and a potential therapeutic target in FOLFIRINOX regimens resistance. Colony formation assays and flow cytometry assays were performed to explore the roles of *GALNT5* in cell proliferation and apoptosis in PDAC treated with FOLFIRINOX. IC50 alterations were calculated in *GALNT5* knockdown and overexpressed cell lines. RNA-seq followed by GSEA (gene set enrichment analysis) was displayed to explore the potential mechanism. WB (western blotting), real-time PCR, and IF (immunofluorescence) were performed to validate relative pathways. The mouse orthotopic xenograft PDAC model was established to examine *GALNT5* functions in vivo. *GALNT5* was highly expressed in PDAC tissues and predicted poor prognosis in PDAC. Upregulation of *GALNT5* in PDAC cells conferred FOLFIRINOX resistance on PDAC by inhibiting DNA damage. Moreover, *GALNT5* interacted with *MYH9*, thus participating in the activation of the NOTCH pathways, resulting in hampering FOI-induced DNA damage. Functions of *GALNT5* promoting FOLFIRINOX resistance were validated in vivo. In this study, we found that aberrantly overexpressed *GALNT5* in PDAC took part in the activation of the NOTCH pathway by interacting with *MYH9*, thus inhibiting the DDR to achieve FOLFIRINOX resistance and causing poor prognosis. We identified GALNT5 as a potential therapeutic target for PDAC patients resistant to FOLFIRINOX chemotherapy.

## Introduction

Pancreatic cancer is one of the most aggressive malignant tumors, with its incidence ranking 8th among malignant tumors in China and being the 7th leading cause of cancer deaths globally, with pancreatic ductal adenocarcinoma projected to become the 2nd leading cause of cancer deaths in the next 20 years [1, 2]. Traditional treatments for pancreatic cancer mainly include surgical resection, chemotherapy, and radiotherapy. For resectable pancreatic cancer, surgery is the preferred treatment option. However, accounts for pancreatic cancer are often accompanied by lymph node invasion or distant organ metastasis at an early stage, less than 20% of patients have the opportunity for surgical treatment at the time of diagnosis [3]. For borderline resectable (BR) and locally advanced (LA) pancreatic cancer, the aim is to turn them into resectable pancreatic cancer by means such as chemotherapy to strive for surgical treatment. For pancreatic cancer that cannot be resected surgically, chemotherapy and radiotherapy are currently the main treatments. At present, first-line chemotherapy regiments for pancreatic cancer mainly include gemcitabine-based regiments such as AG (albumin paclitaxel and gemcitabine) and fluorouracil-based regiments such as FOLFIRINOX (5-fluorouracil, oxaliplatin, Irinotecan).

The FOLFIRINOX regimen was first studied clinically in patients with metastatic pancreatic cancer since 2003 [4]. With the emergence of a large number of findings, in recent years the NCCN (National Comprehensive Cancer Network) guidelines have recommended the FOLFIRINOX regimen as the standard first-line chemotherapy regimen for locally advanced and metastatic pancreatic cancer. The findings that modified FOLFIRINOX versus gemcitabine adjuvant chemotherapy after resection of pancreatic cancer, as reported by ASCO in 2018 [5], have further extended this three-agent combination chemotherapy regimen to postoperative adjuvant chemotherapy. FOLFIRINOX regimens are further recommended by the new NCCN guidelines as a first-line adjuvant chemotherapy option for patients in good physical condition. The intervention of chemotherapy has effectively improved the outcome of pancreatic cancer surgery and the prognosis of patients [6]. Furthermore, a number of studies have reported that FOLFIRINOX regimens result in significantly longer survival than gemcitabine in patients undergoing pancreatic cancer resection [5, 7], but it has to face the frequent occurrence of drug resistance. Research into the mechanism of resistance to gemcitabine is currently being intensified, but there has been little research on the mechanism of resistance to other first-line chemotherapy regimens such as FOLFIRINOX.

Mechanisms underlying chemotherapy resistance could be summarized in several aspects, including reduced drug activation, increased drug inactivation, increased drug excretion, reduced drug uptake and delivery, and escape from apoptosis et al. [8]. PDAC (Pancreatic ductal adenocarcinoma) therapy is often confronted with chemotherapy resistance, accounting for dense desmoplastic stroma, immune-suppressed tumor microenvironment, and high occurrence of gene mutations such as K-ras mutant and TP53 mutant. Current chemotherapy regimens for PDAC mainly depended on gemcitabine-based or fluorouracil-based treatments, with a small number of platinum-based treatments. The mechanism of gemcitabine resistance is the most studied in PDAC. For example, previous reported work has demonstrated that gemcitabine resistance enhanced with p53 degradation, which upregulated pyrimidine biosynthesis and alleviated replication stress [9]. Besides, gemcitabine uptake and delivery were attenuated, accounting for the inhibition of expression of the gemcitabine transporter ENT1 [10]. Moreover, a CAF (cancer-associated fibroblasts)-specific circRNA, circFARP1 enables CAF to promote gemcitabine resistance through LIF/STAT3 axis [11]. With regard to platinum-based chemotherapeutic agents in PDAC, studies have been mostly limited to patients with BRCA mutations. For these patients, in addition to platinum drugs, PARP inhibitors such as Olaparib, are often adopted to assist treatment. Furthermore, homologous recombination (HR) proficiency and secondary mutations that restored partial functionality were identified as the leading cause of platinum-based resistance [12]. Unfortunately, little research on the mechanisms of fluorouracil-based regimen resistance has been achieved, except for the reports in 2021 that FOLFIRINOX resistance was upregulated by the microRNA MIR1307 [13].

The GALNT family consists of 20 members, which are mainly involved in glycosyltransferase activity, carbohydrate binding, and metal ion binding activity in cells. Many members have been confirmed to influence tumorigenesis and development in different cancer species [14–17], demonstrating the potential and value of the family in cancer research. Meanwhile, it is worth noting that *GALNT5*, which is hardly ever reported in PDAC, is the only member in this family that is highly expressed in pancreatic cancer and has a worse prognosis compared with the low-expression group.

This study found that *GALNT5* plays a key role in FOLFIRINOX chemotherapy resistance. *GALNT5* is significantly upregulated in PDAC tissues compared to adjacent normal tissues and correlated with poor prognosis. It was confirmed in vitro and in vivo that *GALNT5* affects the resistance of the FOLFIRINOX chemotherapy regimen mainly through oxaliplatin resistance. *GALNT5* silencing enhances the DNA damage repair pathway (DDR) and finally confers the sensitivity of FOLFIRINOX chemotherapy on PADC

## Results

### Expression pattern and clinical relevance of GALNT5 in PDAC

**Fig. 1:**
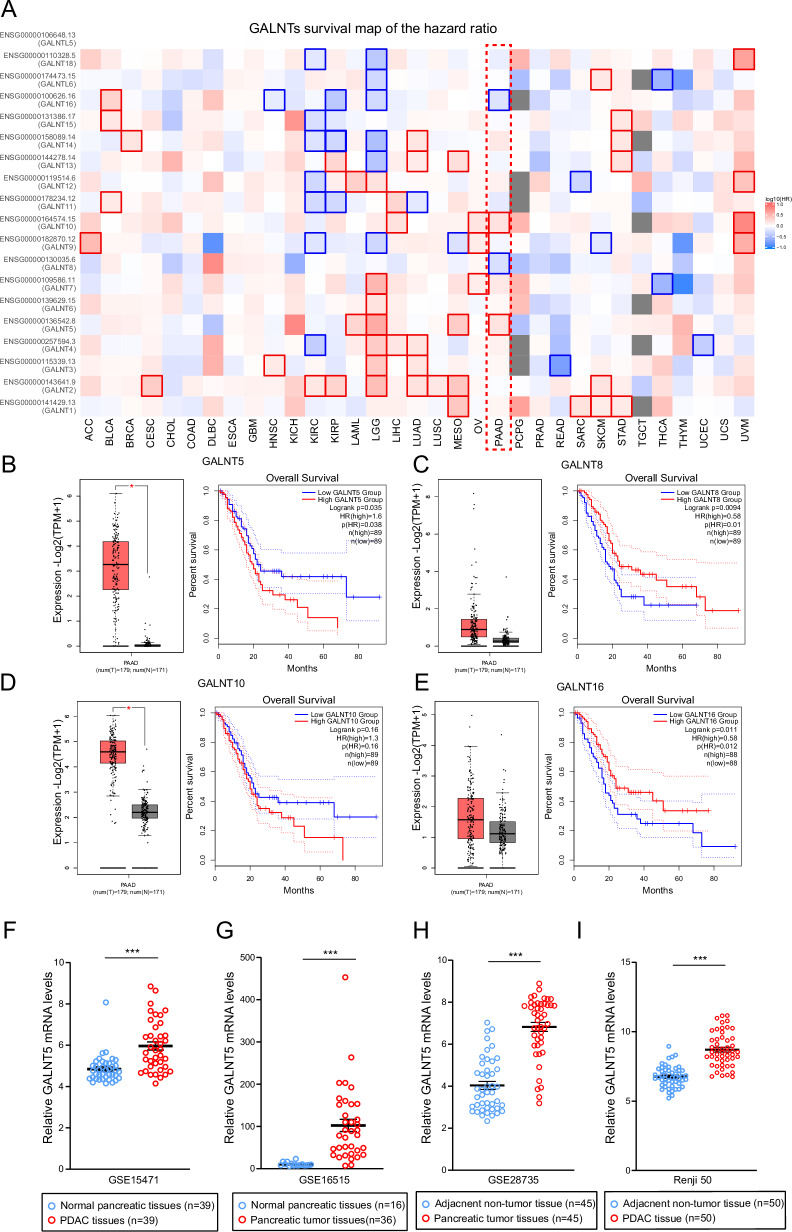
Correlation analysis of expression levels and prognostic risk of members of the GALNT family in pan-carcinoma based on public databases. **A** Analysis of prognosis of pan-cancer according to expressions of different GALNT family members (TCGA and GTXs datasets). **B–E** Individual expression and prognosis of GALNT5 (**B**), GALNET8 (**C**), GALNT10 (**D**), and GALNT16 (**E**) in TCGA and GTXs (*n* = 350) (Two-tailed unpaired Student t-test, ***p < 0.001). **F** GALNT5 expressions analysis in PDAC tissues and matching normal pancreatic tissues in GEO datasets GSE15471 (Two-tailed unpaired Student t-test, ***p < 0.001). **G** GALNT5 expression difference between the pancreatic tumor and normal samples in GEO datasets GSE16515 (Two-tailed unpaired Student t-test, ***p < 0.001). **H** GALNT5 expression profile of the paired pancreatic tumor and adjacent non-tumor tissues in GEO datasets GSE28735 (Two-tailed unpaired Student t-test, ***p < 0.001). **I** GALNT5 expression of PDAC tissue and paired adjacent non-tumor tissue in GEO datasets Renji cohorts / GSE102238 (Two-tailed unpaired Student t-test, ***p < 0.001).

**Fig. 2:**
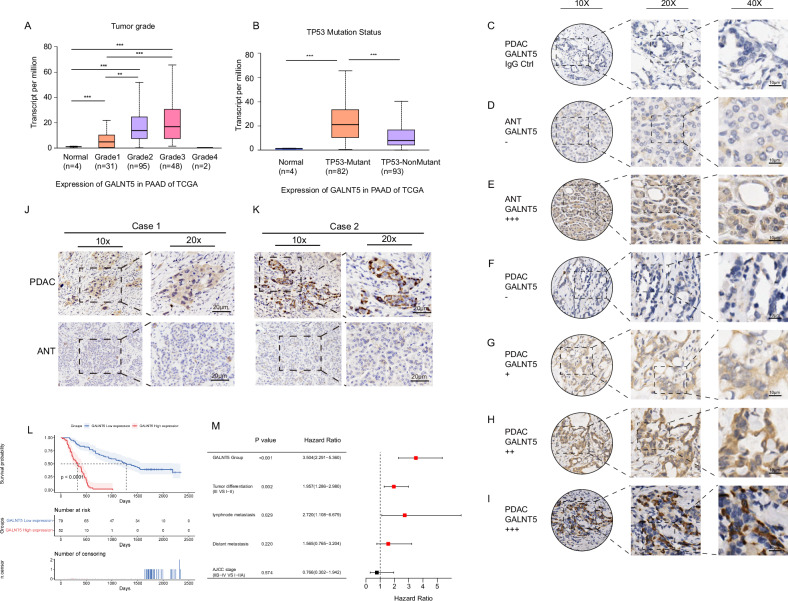
The expression pattern and clinical significance of GALNT5 in pancreatic cancer. **A** TCGA database demonstrating the expressions of *GALNT5* in different grades (Normal, *n* = 4; Grade1, *n* = 31; Grade1, *n* = 95; Grade2, *n* = 48; Grade4, *n* = 2) of PDAC (Two-tailed unpaired Student t-test, ***p < 0.001). **B** TCGA database showing the relationship of *GALNT5* with *TP53* mutation in PDAC (Normal, *n* = 4; *TP53*-Mutant, *n* = 82; *TP53*-NonMutant, *n* = 93, Two-tailed unpaired Student t-test, ***p < 0.001). **C–I** IHC-P staining assessing the expression and distribution of *GALNT5* in Renji PDAC TMA (*n* = 150) and scoring according to intensity (Three fields assessed per tissue, scale bar: 40um, 20um, 10um). **J–K** IHC-P staining displaying that *GALNT5* is highly expressed in PDAC compared with matched adjacent non-tumor tissues (Three fields assessed per tissue, scale bar: 20um). **L** Kaplan-Meier analysis showing overall survival and median survival time of TMA. **M** Multivariate COX regression analysis unraveling the independent risk factors of TMA corresponding clinical index. Significant difference *P* <0.05.

To investigate which members of the GALNT family contribute to the prognosis of pancreatic cancer, we analyzed the correlation between the expressions of GALNT family members and the overall survival of pancreatic cancer in TCGA (The Cancer Genome Atlas Program) and GTXs (Genotype-Tissue Expression) via GEPIA (Fig. 1A). The survival map of the hazard ratio showed that only the expressions of *GALNT5*, *GALNT8*, *GALNT10*, and *GALNT16* displayed significant differences in prognosis with pancreatic cancer. High expressions of *GALNT5* and *GALNT10* are positively correlated with pancreatic cancer survival, while expressions of *GALNT8* AND *GALNT10* are negatively correlated with survival in PAAD (pancreatic adenocarcinoma). We further explored them respectively in TCGA combined with GTXs and found that only *GALNT5* has significantly high expression in tumor tissues compared with adjacent normal tissues and highly expressed *GALNT5* suggests a worse prognosis in PAAD (Fig. 1B-E). We validated its expression in four Gene Expression Omnibus (GEO) datasets: GSE15471, GSE16515, GSE28735, and Ren Ji cohort (GSE102238) (Fig. 1F-I) and confirmed that GALNT5 was more highly expressed in pancreatic cancer tissues. Next, we analyzed some clinical events and several common gene mutants in PAAD of TCGA datasets based on *GALNT5* expression via UALCAN (The University of Alabama at Birmingham Cancer Data Analysis Portal). We found that *GALNT5* expression increased with increasing tumor grade (Fig. 2A), but not with individual cancer stage and nodal metastasis (Supplementary Fig. S1). Besides, aberrantly upregulated *GALNT5* accompanied by more TP53-Mutant (Fig. 2B). To validate the clinical relevance of *GALNT5* in PDAC, we performed immunohistochemical (IHC) staining in pancreatic tumor TMA (tissue micro-assays) containing 150 pathology-verified PDAC specimens with paired corresponding adjacent pancreatic tissues from the Ren Ji cohort and scored the TMA based on the strength and range of IHC staining. Furthermore, we analyzed several clinical indexes based on the IHC staining scores and found that the upregulation of *GALNT5* was correlated with poor prognosis (Fig. 2K). Moreover, *GALNT5* was significantly related to tumor differentiation (III VS I-II), and lymph node metastasis, without relation to distant metastasis and AJCC stage (Fig. 2M). In summary, *GALNT5* may play a role in PDAC progression.

### GALNT5 confers chemotherapy resistance of FOLFIRINOX on PDAC

**Fig. 3:**
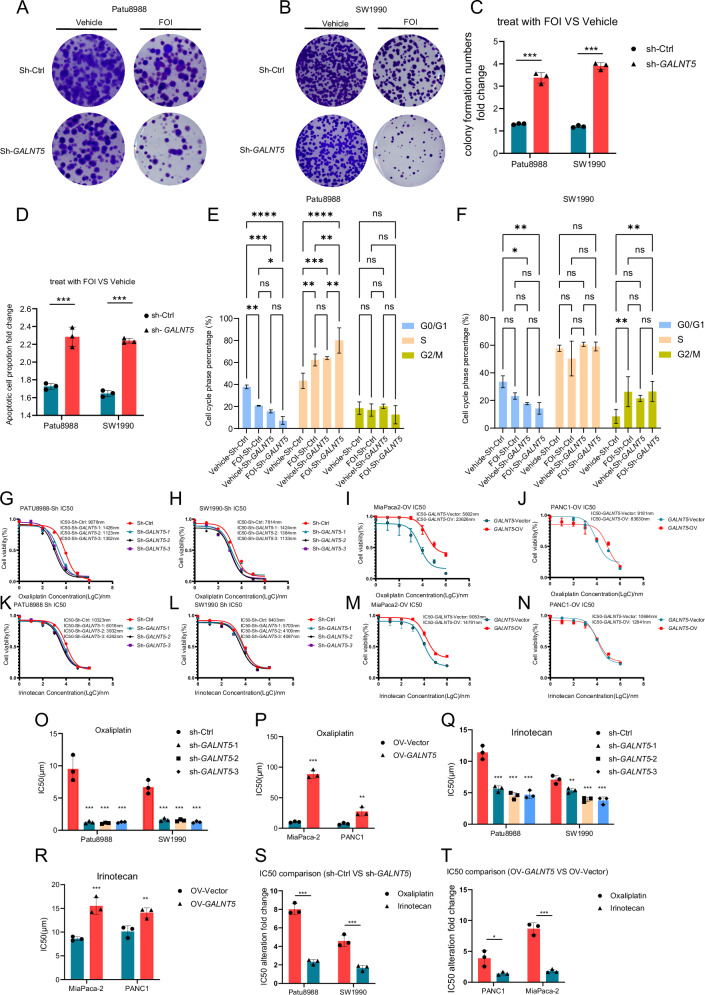
GALNT5 affects the chemotherapy sensitivity of FOLFIRINOX regimen and its components in vitro. **A–C** Colony formation assays evaluating the proliferation difference of Patu8988 (**A**) and SW-1990 (**B**) experienced RNAi of *GALNT5* treated with or without FOI (Three fields assessed per dish, three individual biological replicates performed, Two-way ANOVA, ***p < 0.001). FOI: the combination of 5-FU (4um), oxaliplatin (10um), and Irinotecan (10um) based on IC50 in WT PDAC cells, respectively. **D** Flow cytometry revealing the apoptosis difference of Patu8988 and SW-1990 experienced RNAi of *GALNT5* treated with or without FOI (Three individual biological replicates performed, Two-way ANOVA, ***p < 0.001). (**E-F**) Flow cytometry revealing the cell cycle phase alteration difference of (**E**) Patu8988 and (**F**) SW-1990 experienced RNAi of *GALNT5* treated with or without FOI (Three individual biological replicates performed, Two-way ANOVA, ***p < 0.001). **G–J** Oxaliplatin dose-response relationship curves were drawn and IC50 of oxaliplatin in the *GALNT5* knockdown cell line (**G**) PATU8988 and (H)SW1990 and overexpression cell line (**I**) MiaPaca-2 and (**J**) PANC1 were calculated. **K–N** Oxaliplatin dose-response relationship curves were drawn and IC50 of oxaliplatin in the *GALNT5* knockdown cell line (**K**) PATU8988 and (**L**) SW1990 and overexpression cell line (**M**) MiaPaca-2 and (**N**) PANC1 were calculated. **Q–R** statistics analysis of IC50 differences demonstrating IC50 alterations of (O-P) oxaliplatin and **Q**–**R** Irinotecan when GALNT5 was knocked down or overexpressed. (Three individual biological replicates performed, Two-way ANOVA, ***p < 0.001). **S–T** IC50 alteration fold change showing differences between degrees of oxaliplatin and Irinotecan IC50 in cell lines treated with *GALNT5* knockdown and overexpression. (Three individual biological replicates performed, Two-way ANOVA, ***p < 0.001).

We referred to the previously reported work that microRNA MIR1307 modulates PDAC cell sensitivity to FOLFIRINOX [13] and decided to simulate FOLFIRINOX chemotherapy regimens via the combination (FOI) of 5-fluorouracil, oxaliplatin, and Irinotecan based on respective IC50. To explore whether *GALNT5* affects FOLFIRINOX resistance, we selected SW-1990 and Patu8988 cell lines to perform *GALNT5* knockdown (sh-*GALNT5*) and PANC1 and MiaPaca-2 cell lines to perform *GALNT5* overexpression (OV-*GALNT5*) depending on the baseline of *GALNT5* expression level. (supplementary Fig. S2). Colony formation experiments were displayed (Fig. 3A-C) to investigate the role of *GALNT5* in PDAC to FOLFIRINOX sensitivity. We calculated the declined degrees between colonies treated with FOI and Vehicle, and observed more inhibition of cell proliferation in Patu8988 and SW-1990 sh-*GALNT5*, indicating that knockdown of *GALNT5* made PDAC cells more sensitive to FOI. Moreover, we performed flow cytometry (Fig. 3D) to examine the alterations of sensitivity to FOLFIRINOX in SW-1990 and Patu8988 cell lines. We also calculated the increased degree of apoptosis in cells treated with FOI compared with Vehicle and found more apoptosis in Patu8988 and SW-1990 sh-*GALNT5*, demonstrating knockdown *GALNT5* made PDAC cells more susceptible to FOI. We then set up the same experimental grouping conditions and detected changes in the cell cycle. We found that GALNT5 knockdown treatment alone or FOI treatment alone could promote the decrease of the proportion of G0/G1 phase cells, and this phenomenon was more obvious when the two treatments were given at the same time, mainly because GALNT5 knockdown treatment and FOI treatment caused significant S phase or G2/M phase arrest of pancreatic cancer cells (Fig. 3E-F) In summary, *GALNT5* confers FOI resistance on PDAC cells.

Furthermore, we would like to confirm whether *GALNT5*’s promotion of the FOLFIRINOX resistance significantly affects one of its components. We calculated the IC50 of oxaliplatin (Fig. 3G-J, O, P), irinotecan (Figs. 3K-N, Q, R), and 5-FU (Supplementary, Fig. S2) in *GALNT5* knockdown and overexpression cell lines respectively. We observed that knockdown and overexpression of *GALNT5* mainly changed the sensitivity to oxaliplatin and partially to irinotecan (Fig. 3S-T), while no significant effects were found for 5-FU (Supplementary, Fig. S3). These results suggest that *GALNT5* may influence overall chemotherapy sensitivity by affecting the platinum components of the FOLFIRINOX regimen.

### GALNT5 promotes FOLFIRINOX resistance by attenuating DNA damage

**Fig. 4:**
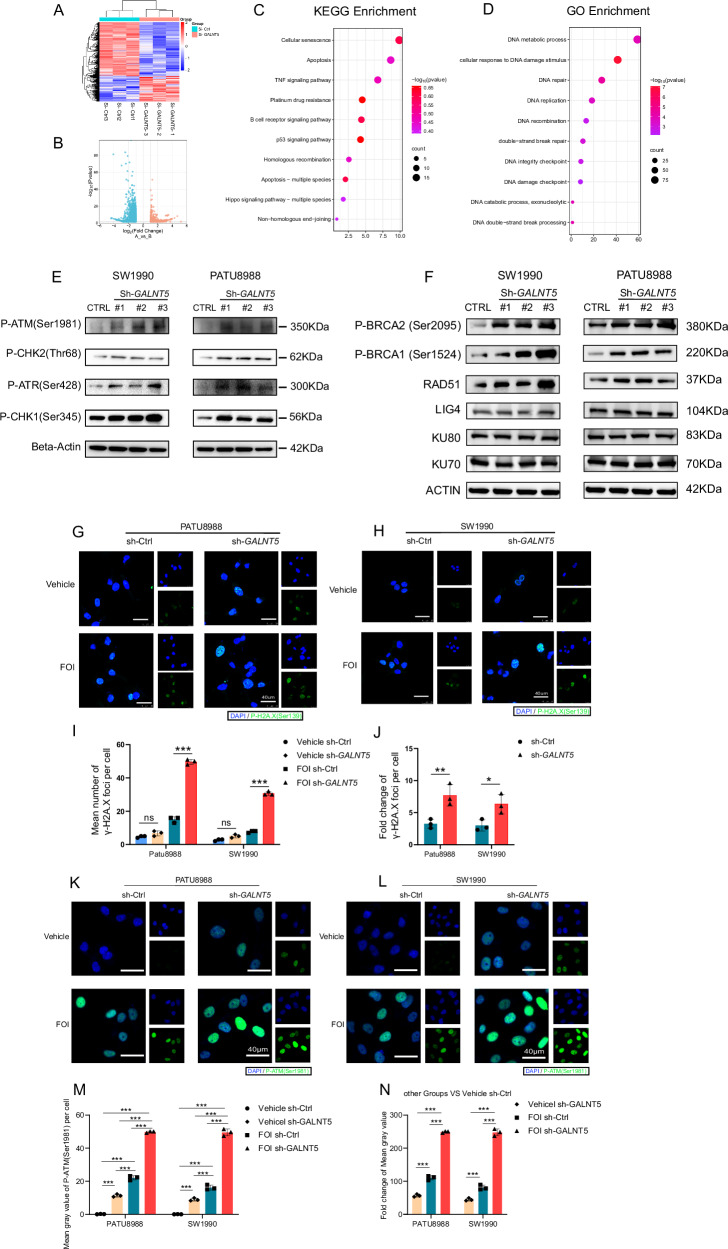
GALNT5 affects the DDR pathway, especially the homologous recombination repair pathway in the DSB in vitro. **A, B** heatmap and volcano plot showing the alteration of gene expression after the knockdown of *GALNT5* in the Patu8988 cell line (│log2FC│≥1 *p* < 0.05). **C–D** Different pathway analyses were displayed on RNA-seq utilizing KEGG enrichment and GO enrichment in *GALNT5*-knockdown cells. **E** WB analyzing the alterations of DNA damage pathway in Patu8988 and SW-1990 cell line after GALNT5-knockdown. **F** WB analyzing the alterations of HR and NHEJ pathway in Patu8988 and SW-1990 cell line after GALNT5-knockdown. **G–J** GALNT5 knockdown cells were treated with DMSO or FOI for 24 h and assessed for phosphorylated γ-H2AX staining by immunofluorescence. Foci numbers of γ-H2AX were counted for the measurements of DNA damage. (Scale bar, 40 μm. Three fields were assessed per group. Three individual biological replicates were performed, Two-way ANOVA, ns *p* > 0.05; *p < 0.05; **p < 0.01; ***p < 0.001). **K–N** GALNT5 knockdown cells were treated with DMSO or FOI for 24 h and assessed for phosphorylated ATM (Ser1981) staining by immunofluorescence. Mean gray value of P-ATM (Ser1981) were counted for the measurements of DSB damage. (Scale bar, 40 μm. Three fields were assessed per group. Three individual biological replicates were performed, Two-way ANOVA, ns *p* > 0.05; *p < 0.05; **p < 0.01; ***p < 0.001).

To investigate the underlying mechanisms of *GALNT5* promoting FOLFIRINOX resistance, we performed mRNA-seq analysis on sh-Ctrl cells and sh-*GALNT5* cells. Silencing *GALNT5* remarkably changed the gene expression signature (Fig. 4A). Differentially expressed genes (DEGs) showed that 1197 genes were downregulated and 582 genes were upregulated (Fig. 4B, Supplementary table. 3), indicating that *GALNT5* is involved in many cellular activities. KEGG enrichment analysis of DEGs (Fig. 4C) significantly enriched platinum drug resistance and homologous recombination, while GO enrichment analysis of DEGs (Fig. 4D) revealed that the most affected molecular functions were the cell cycle and cellular response to DNA damage stimulus. Therefore, we hypothesized that *GALNT5* promotes FOLFIRINOX resistance primarily by downregulating platinum-associated DNA damage. To examine whether DNA damage repair pathways were influenced by *GALNT5*. We performed western blotting and found that phosphorylated ATM, CHK2, ATR, and CHK1 were enhanced (Fig. 4E) when silencing *GALNT5*, indicating that *GALNT5* attenuated DNA damage.

Considering that the types of DNA damage caused by platinum-containing chemotherapy are mainly DNA double-strand breaks, and the DNA repair modes in response to DNA double-strand break damage are mainly HR (Homologous Recombination) repair and NHEJ (non-homologous end-joining) repair, we further examined the changes of key molecules in the two pathways after knocking down GALNT5. It was found that the expression of P-BRCA1 (Ser2095), P-BRCA1 (Ser1524) and RAD51 related to HR repair was significantly changed after knocking down GALNT5, while the expression of KU70, KU80, LIG4 related to NHEJ repair was not significantly changed after knocking down GALNT5. Therefore, we suggest that GALNT5 may affect FOLFIRINOX chemotherapy sensitivity in pancreatic cancer cells primarily by affecting the HR repair pathway during DDR (Fig. 4F) Considering that HR repair occurs mainly between the S or G2 phases of the cell cycle, this result is consistent with our previous detection of cell cycle changes (Fig. 3E-F).

Immunofluorescence to stain γH2A.X was further performed in Patu8988 sh-*GALNT5* (Fig. 4G) and SW-1990 sh-*GALNT5* (Fig. 4H). We measured the alteration of DNA damage in cells treated with FOI and Vehicle according to the staining foci number fold changes per cell (Fig. 4I-J) and found that more DNA damage was induced in sh-*GALNT5*(Fig. 4H-I). These results revealed that *GALNT5* is associated with platinum resistance which may be the cause of FOLFIRINOX resistance and promotes FOLFIRINOX resistance by attenuating DNA damage. In order to further detail the types of DNA damage involved in this study, P-ATM (Ser1981) immunofluorescence staining was performed under the same experimental grouping conditions and a consistent conclusion was obtained (Fig. 4K-N). Since ATM is one of the most important molecules involved in the repair of DNA double-strand break damage, combined with the previous conclusion that “platinum components may be the primary cause of FOLFIRINOX chemotherapy resistance”, we believe that the types of DNA damage caused by GALNT5 are mainly DNA double-strand break damage (DSB). In summary, the potential mechanism by which GALNT5 affects FOLFIRINOX chemotherapy resistance in pancreatic cancer may be that it affects the HR repair process of DSB during chemotherapy.

### GALNT5 interacts with MYH9 and activates the NOTCH pathway

**Fig. 5:**
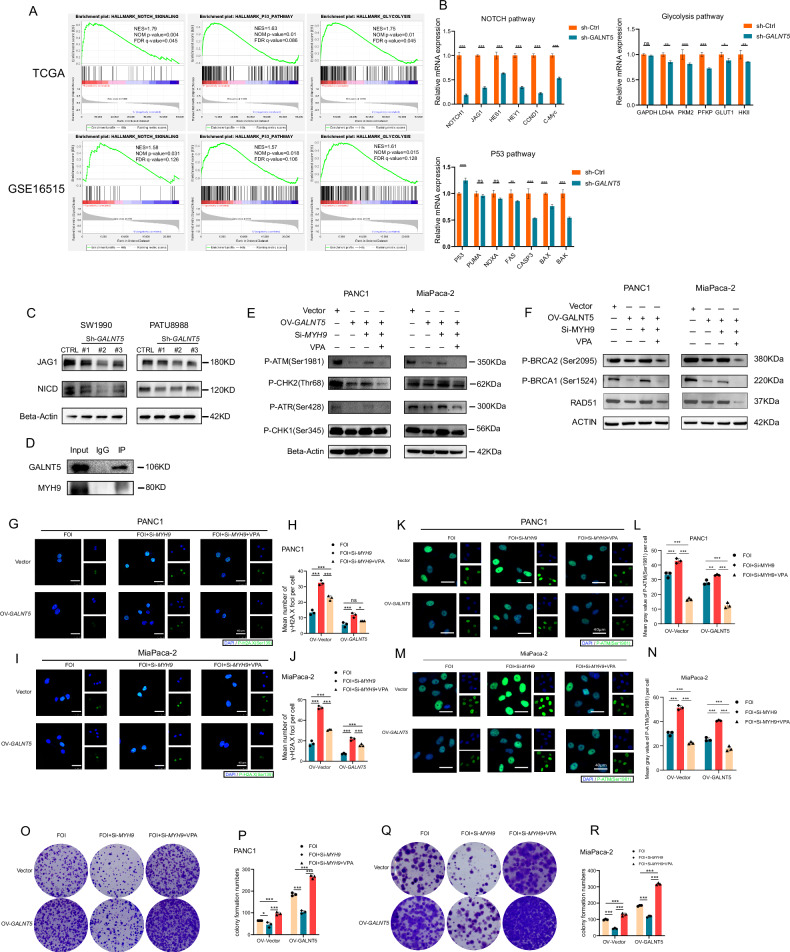
GALNT5 up-regulates NOTCH pathway by interacting with MYH9 in vitro to promote the activation of DDR pathway, especially HR repair pathway. **A** GSEA was performed on TCGA datasets (upper) and GEO datasets GSE16515 (lower) based on GALNT5 expression. **B** Real-time PCR examining the mRNA expressions of the NOTCH pathway target gene, the key enzymes in the glycolysis pathway, and key genes in P53 pathways (Two-tailed unpaired Student t-test, ns *p* > 0.05; ***p < 0.001). **C** Co-immunoprecipitation of *GALNT5* and *MYH9*. **D** WB analysis showing JAG1 and NICD downregulated when knockdown GALNT5. **E–F** WB analysis of Phosphorylated ATM, CHK2, ATR, CHK1 illustrating alteration of integrated DNA damage and Phosphorylated BRCA1, BRCA2 and RAD51 illustrating alteration of HR pathway in DSB damage in PANC1 and MiaPaca-2 cell line treated with *GALNT5* overexpression, RNAi of *MYH9* and the NOTCH pathway agonist valproic acid (VPA). **G**–**L** Immunofluorescence of Phosphorylated γH2A.X was performed in PANC1 and MiaPaca-2 cell lines and mean numbers of IF foci were calculated to measure the alteration of DNA damage in PDAC cells. (Three individual biological replicates were performed, Two-way ANOVA, ns *p* > 0.05; *p < 0.05; ***p < 0.001). **I–N** Immunofluorescence of Phosphorylated ATM (Ser1981) was performed in PANC1 and MiaPaca-2 cell lines and mean gray value of P-ATM (Ser1981) were calculated to measure the alteration of DSB damage in PDAC cells. (Three individual biological replicates were performed, Two-way ANOVA, ns *p* > 0.05; *p < 0.05; ***p < 0.001). **O–R** Colony formation assays were adopted to validate the function of MYH9 and the NOTCH pathway in cell proliferation. (Three individual biological replicates were performed, Two-way ANOVA, *p < 0.05; ***p < 0.001).

To further investigate the mechanisms of *GALNT5* promoting FOLFIRINOX resistance, gene set enrichment analysis (GSEA) was performed among the samples of TCGA datasets and GEO datasets GSE16515 (Fig. 5A, Supplementary Fig. S4). We found that only the NOTCH signaling pathway, P53 signaling pathway, and glycolysis-associated pathway were enriched in both “HALLMARK” and “KEGG” gene set databases. In order to figure out whether these pathways are affected. We knocked down *GALNT5* in PDAC cells and detected the changes in the target genes of the NOTCH pathway, the key enzymes in the glycolysis pathway, and key genes in the P53 pathways by real-time PCR (Fig. 5B). It turned out that the target genes of the NOTCH pathway were changed most pronounced. Therefore, we ultimately chose the NOTCH pathway for further exploration. First, we validated the alteration of the NOTCH pathway after silencing GALNT5 and found that NICD and JAG1 were significantly downregulated (Fig. 5C), suggesting that *GALNT5* took part in the activation of the NOTCH pathway. Next, mass spectrometry was performed to filtrate the potential candidate with which *GALNT5* interacted. Given that *MYH9* had the highest sum pep score in mass spectrometry results (Supplementary Table. 4) and has been reported to positively regulate the NOTCH pathway [18], we finally selected *MYH9* as a candidate for mediating between *GALNT5* and the NOTCH pathway. The interaction between *GALNT5* and *MYH9* was confirmed by CO-Immunoprecipitation (Fig. 5D). We further studied the role of *MYH9* in *GALNT5*-mediated DNA damage. We performed WB in PANC1 and MiaPaca-2 OV-*GALNT5* cell lines and observed that DNA damage was reduced by overexpression of *GALNT5* and the trend rebounded after silencing *MYH9*. Furthermore, rebounded DNA damage induced by the knockdown of *MYH9* was further rescued by the addition of the NOTCH pathway agonist valproic acid (VPA) (Fig. 5E). Besides, IF staining of P-γH2A.X was performed (Fig. 5G-I) in the same groups and we obtained the consistent result (Fig. 5H-J). Changes in HR repair pathways and DSB are consistent with previous conclusions (Fig. 5F, Fig. 5K-N). Finally, we examined the role of *MYH9* in cell proliferation via colony formation experiments in PANC1 (Fig. 5O) and MiaPaca-2 (Fig. 5Q) cell lines. We found that the knockdown of *MYH9* inhibited cell proliferation and the trend was rescued with the intervention of VPA (Fig. O-R). These results suggest that *GALNT5* positively regulates the NOTCH pathway by interacting with *MYH9*, thereby reducing DNA damage and promoting FOLFIRINOX resistance.

### Validation of the role of GALNT5 in chemoresistance in vivo

**Fig. 6:**
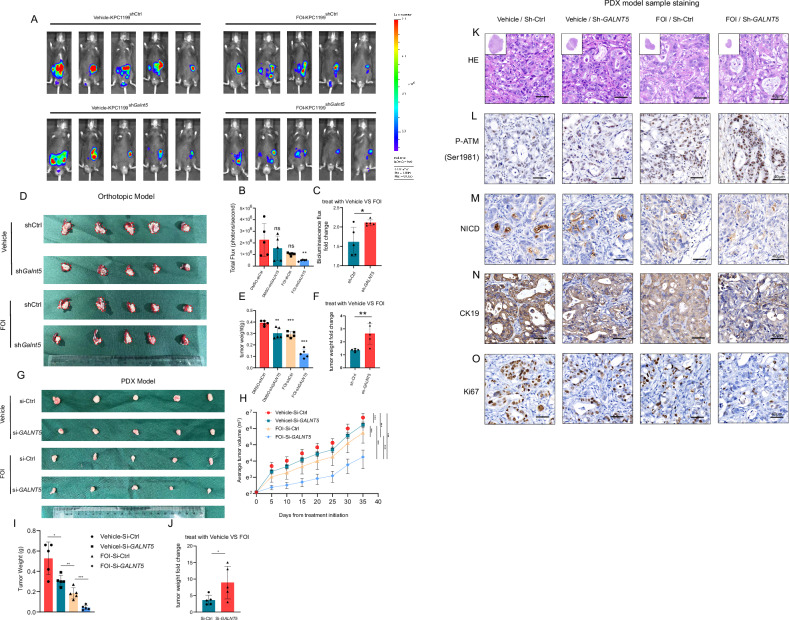
High expression of GALNT5 up-regulates the sensitivity of pancreatic cancer to FOLFIRINOX chemotherapy in vivo. **A–C** KPC1199 cells expressing luciferase were implanted in pancreases in situ. **A** Bioluminescence images of PDAC mouse orthotopic xenograft mode were taken to assess alterations of cell proliferation. **B** Analysis of total flux emission differences revealing a decreased tumor size after knocking down GALNT5 and treated with FOI in vivo. **C** Bioluminescence flux fold change differences were calculated to evaluate the role of GALNT5 in FOI sensitivity for PDAC cell proliferation. (Two-tailed unpaired Student t-test, ***p < 0.001). **D–F** orthotopic xenograft tumors were taken and weighed, showing that treated with FOI and the knockdown of GALNT5 inhibited the growth of PDAC in vivo and the knockdown of GALNT5 conferred FOI sensitivity on PDAC in vivo. ((*n* = 5 mice per group, mean± s.e.m.; two-tailed unpaired t test). *P* < 0.05; **P < 0.01; ***P < 0.001.). **G** Representative images of PDX tissues, which were formed by knocking down GALNT5 and treated with FOI in vivo. **H** The volume of indicated tumors was measured on the indicated days. **I–J** The weight of indicated PDX tissues were measured after tumor excision and the difference of fold changes of tumor weights were calculated. **G** HE staining and IHC staining of P-ATM (Ser1981), NICD, Ki67 and CK19 in PDX tissue samples. (Three fields assessed per tissue, scale bar: 40 um).

To investigate the role of *GALNT5* in FOI resistance in vivo, we adopted KPC1199 cells to establish a mouse orthotopic xenograft PDAC model. KPC1199 cells derived from the KPC mouse model which contains K-ras^LSL.G12D/+^; and Trp53^R172H/+^, thus restored the common gene mutation of PDAC. Bioluminescence images (Fig. 6A) were taken to assess the tumor volume alterations difference between the groups treated with FOI and Vehicle in vivo. We measured the total flux and found that the emission decreased more in the sh-*GALNT5* groups (Fig. 6B-C), indicating that the knockdown of *GALNT5* made PDAC cells more susceptible to FOI. Furthermore, we took out orthotopic xenograft tumors (Fig. 6D) and analyzed their weight (Fig. 6E). We found that sh-*GALNT5* groups showed more growth inhibition when treated with FOI, compared with the sh-Ctrl groups (Fig. 6F). The volume and weight analysis of mouse PDX pancreatic cancer model obtained the same results as that of the orthotopic xenograft model (Fig. 6G-J) We further carried out HE and histochemical staining on PDX model samples, and the results showed that: the PDX tissue differentiation in the Vehicle-ShCtrl group was the lowest, the PDX tissue differentiation was improved after FOI treatment alone or GALNT5 knockdown, and the group FOI- ShGALNT5 receiving FOI treatment and GALNT5 knockdown at the same time had the highest degree of differentiation (Fig. 6K). The trend change of DNA damage in PDX tissue reflected by P-ATM (Ser1981) staining was consistent with that of HE staining (Fig. 6L). The trend of PDX tumor growth reflected by the histochemical staining changes of NICD, Ki67, and CK19 (Fig. 6M-O) was also consistent with the above results. These results show that GALNT5 knockdown and FOI treatment have a synergistic effect on inhibiting the growth and differentiation of pancreatic cancer, suggesting that GALNT5 knockdown can make pancreatic cancer cells more sensitive to FOLFIRINOX chemotherapy

### Validation of GALNT5-MYH9-NICD interaction on chemotherapy resistance in vivo

**Fig. 7:**
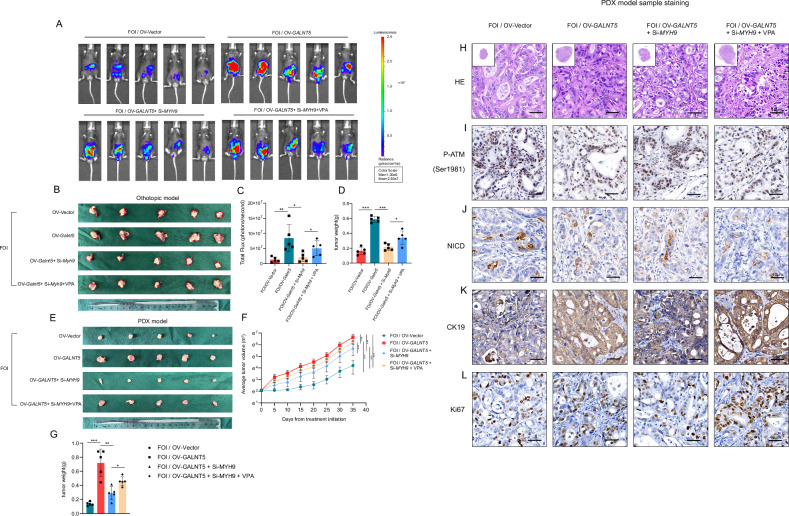
GALNT5 upregulates the NOTCH pathway by binding to MYH9 in vivo, conferring chemotherapy resistance to FOLFIRINOX in pancreatic cancer. **A, C** KPC1199 cells expressing luciferase were implanted in pancreases in situ and all groups were treated with FOI. **A** Bioluminescence images of PDAC mouse orthotopic xenograft mode were taken to assess alterations of cell proliferation. **C** Analysis of total flux emission differences. **B, D** Representative images of tumors, which were formed by empty vector- or overexpressing GALNT5 (OV-GALNT5), or overexpressing GALNT5 and knockdown of MYH9 (OV-GALNT5+Si-MYH9), or additionally treated with NOTCH1 signaling agonists (OV-GALNT5+Si-MYH9 + VPA). orthotopic xenograft tumors were taken and weighed ((*n* = 5 mice per group, mean± s.e.m.; two-tailed unpaired t test). *P* < 0.05; **P < 0.01; ***P < 0.001). **E** Representative images of PDX tissues, which were formed by knocking down GALNT5 and treated with FOI in vivo. **F** The volume of indicated tumors was measured on the indicated days. **G** The weight of indicated PDX tissues were measured after tumor excision and the difference of fold changes of tumor weights were calculated. **H–L** HE staining and IHC staining of P-ATM (Ser1981), NICD, Ki67, and CK19 in PDX tissue samples.

To verify the interaction of the GALNT5-MYH9-NICD axis in vivo, we used the same mouse KPC1199 cell implanted orthotopic xenograft tumor model and the mouse pancreatic cancer PDX model as in the above experiment, and all models were treated with FOI once a week to restore the clinical administration status. Our results suggested that in the context of FOI treatment, compared with the vector group, the tumor was significantly enlarged after overexpression of GALNT5, while the tumor growth was significantly inhibited after continued knocking down MYH9. This trend was reversed after additional intraperitoneal injection of mouse VPA. We found the same trend in vivo imaging results of mice (Fig. 7A, C), orthotopic xenograft tumor body weight statistics (Fig. 7B, D), and tumor volume statistics (Fig. 7E-F) and weight statistics of the PDX model on day 35 (Fig. 7G). HE and immunohistochemical staining were also performed after sampling the PDX model, and it was observed that: In the OV-Vector group, the differentiation degree was the best after receiving FOI treatment, and the differentiation degree of PDX tissue was significantly reduced after the intratumoral overexpression of GALNT5, while the differentiation degree of PDX tissue was increased again after the intratumoral downgrading of MYH9. Finally, this trend of differentiation was rescued after intraperitoneal injection of VPA (Fig. 7H). The trend change of DNA damage in PDX tissue reflected by P-ATM (Ser1981) staining was consistent with that of HE staining (Fig. 7I). The trend of PDX tumor growth reflected by the histochemical staining changes of NICD, Ki67, and CK19 (Fig. 7J-L)was also consistent with the above results. Combined with previous in vitro results, we suggest that GALNT5 confers chemotherapy resistance to FOLFIRINOX in pancreatic cancer cells by binding to MYH9 and inhibiting NOTCH signaling in vivo.

## Discussion

In this study, we identified *GALNT5* as a potential therapeutic target in PDAC conferring tumor FOLFIRINOX resistance. Especially, we validated in vivo and in vitro that *GALNT5* positively regulates the NOTCH pathway by interacting with *MYH9*, thereby reducing DNA damage and promoting FOLFIRINOX resistance in PDAC. Previous reports showed that *GALNT5* promotes cholangiocarcinoma carcinogenesis and progression through EGFR/AKT/ERK signaling [19, 20]. And *GALNT5* uaRNA (UTR-associated RNA), a lncRNA, which is derived from the 3’-UTR of *GALNT5* was confirmed to interact with HSP90 to promote gastric cancer progression [21]. However, little research has been achieved on *GALNT5* in pancreatic cancer.

FOLFIRINOX regimens are reported to improve the disease-free survival (DFS) and overall survival (OS) of patients with resectable and borderline resectable pancreatic cancer [22–25]. Meanwhile, pancreatic cancer resection and FOLFIRINOX treatment improved the R0 resection (microscopically no residual cancer) rates [26]. A randomized clinical trial from Canada suggested that adjuvant treatment with the modified FOLFIRINOX regimen provided longer overall survival than gemcitabine in patients with pancreatic cancer resection [7]. In another comparative effectiveness cohort study, the FOLFIRINOX regimen was found to provide patients with a longer survival of almost 2 months and fewer posttreatment complications compared with gemcitabine plus nab-paclitaxel [27]. Besides, The FOLFIRINOX was associated with a reduction in the risk of death, with an HR of 0.57 (95% CI, 0.41–0.79 compared with gemcitabine and showed the largest AUC for survival in the curve estimation, followed by gemcitabine plus albumin-bound paclitaxel, gemcitabine plus erlotinib, and GEM [28]. Although FOLFIRINOX is reported superior to the gemcitabine-based chemotherapy regimens in many aspects, the high incidence of toxic side effects and the same problem of drug resistance make this regimen notoriously limited in clinical treatments. Especially, in contrast to the numerous studies on the mechanism of gemcitabine resistance in PDAC, little research on the FOLFIRINOX resistance was achieved. We identified *GALNT5* as a potential FOLFIRINOX resistance marker and a promising therapeutic target. This study revealed that *GALNT5* conferred FOLFIRINOX resistance on PDAC via positive regulation of the NOTCH signaling pathway, which means a combination of FOLFIRINOX and the inhibitor of the NOTCH pathway may significantly reduce the dose of chemotherapy currently required for FOLFIRINOX regimens, thereby alleviating the dilemma of drug resistance.

In this study, we found that *GALNT5* was aberrantly upregulated in PDAC tissue in TCGA datasets, GTXs datasets, GEO datasets, and Renji PDAC cohorts. Moreover, analysis of overall survival based on *GALNT5* expression indicated that highly expressed *GALNT5* predicted a poor prognosis. In addition to exploring the role of *GALNT5* in FOLFIRINOX resistance, colony formation assays and apoptosis flow cytometry reflected the proliferation inhibition of *GALNT5* on PDAC to a certain extent.

Interestingly, analysis of RNA-seq showed that a large number of pathways associated with the cell cycle were inhibited after the knockdown of *GALNT5*, suggesting that *GALNT5* may also promote cell cycle pathways. In general, rapidly proliferating tumor cells are more susceptible to chemotherapy due to increased genomic instability. Nevertheless, PDAC cells did not show more sensitivity to chemotherapy due to the acceleration of the cell cycle induced by abnormally highly expressed *GALNT5*. Instead, we observed an increase in FOLFIRINOX resistance, which meant *GALNT5* balanced PDAC cell proliferation alongside FOLFIRINOX resistance.

Furthermore, the results that *GALNT5* conferred FOLFIRINOX resistance on PDAC mainly via abolishing DNA damage were validated by in vitro and in vivo experiments. DNA damage repair pathways have been validated involving in chemotherapy resistance. Underlying mechanisms of chemotherapy resistance induced by DNA damage response include single-strand break repair, double-strand break repair, and epigenetic control [29]. Platinum resistance is reported to be upregulated by lncRNA UPK1A-AS1 via double-strand break repair [30]. Another work from Israel in 2022 revealed that deficient homologous recombination (HR) conferred platinum sensitivity on PDAC [31]. In addition to platinum-based regimens, gemcitabine-based regimen resistance has been reported to be attenuated via deficient HR, which was induced by the SRSF3-mediated N6-methyladenosine methylation [32]. Besides, previous work reported that SIRT6 inhibitor, 8a could block DNA damage, thus sensitizing PDAC cells to gemcitabine [33]. Although FOLFIRINOX is active in the clinic as a first-line chemotherapy regimen for PDAC, whether the development of its resistance is mediated by DNA damage-related pathways has not been reported in detail. Increased cancer cell stemness induced by cancer stem cells(CSC) in the tumor microenvironment (TME) has been identified as the leading cause of 5-FU resistance [34–36]. As conventional chemotherapy mainly targets highly proliferative and mature cancer cells, CSC survives chemotherapy insults due to its relatively quiescent cell cycle and low degree of differentiation and re-establishes cancer cell numbers. However, our analysis of RNA-seq data and colony formation assays revealed that aberrantly upregulated *GALNT5* in PDAC promoted the cell cycle rather than inhibited it, which laterally indicated that *GALNT5* may not achieve FOLFIRINOX resistance by affecting CSC in TME, and sensitivity of PDAC cells to FOLFIRINOX was mainly affected by *GALNT5* in non-5-FU components. We further proposed that *GALNT5* may mainly promote the resistance of the platinum component in FOLFIRINOX via enhancing DDR, thus promoting FOLFIRINOX resistance in PDAC, instead of the usual 5-FU resistance induced by CSC. This provides us with new insight to improve the situation of FOLFIRINOX resistance that PDAC patients resistant to FOLFIRINOX regimens may benefit from the combination of DDR inhibitors targeting oxaliplatin.

To figure out how DDR was regulated by *GALNT5*, we performed GSEA and finally selected the NOTCH pathways. Alterations in the NOTCH pathway are frequently observed in TME, especially in TME with enhanced immune infiltration and enriched cancer-associated fibroblasts (CAFs). TME includes components that are recruited into the vicinity of the tumor tissue, while in a broader sense, all components that are capable of interacting with tumor cells and influencing tumor progression are part of the TME. The effects of the NOTCH pathway are vital in neuron growth, embryonic development, and cancer, while we have found that it was critical in *GALNT5*-induced drug resistance. Activation of the NOTCH pathway is reported to upregulate the PD-1 expression of CD8 + T cells, thus promoting their exhaustion [37] and the NOTCH pathways have been shown to regulate macrophage maturation towards a tumor-associated macrophage (TAM) phenotype (also known as M2 macrophages) [38, 39]. Besides, the NOTCH pathways induce immunosuppressive myeloid cell recruitment, thus attenuating antitumor immunity [40]. In addition to immune cells, the NOTCH pathways play a role in CAFs. CBF1/suppressor of hairless/LAG1 (CSL) is the key molecular of the NOTCH pathways in the nucleus. CSL no longer represses transcription when the NOTCH pathways are activated. Since many CAFs determinant genes are directly regulated by CSL [41], dysregulation of the NOTCH pathways affects CAFs, thus influencing the proliferation, migration, and invasion of tumor cells [42, 43]. Blockade of the NOTCH pathways was found to reduce the peritumoral desmoplastic reaction in intrahepatic cholangiocarcinoma (iCCA) [44]. Some reports activated NOTCH signaling in CAFs promotes β-catenin-driven radio-resistance and metastasis in DII1+ breast cancer [45]. In this study, GSEA was performed to filtrate potential relative pathways. We observed the most predominant alteration in the NOTCH pathways and further unraveled that *GALNT5* interacted with *MYH9* to positively regulate the NOTCH pathways. Given that alterations in immune infiltration and CAFs are confirmed to contribute to chemotherapy resistance in PDAC [46, 47]. Furthermore, except for functions between the PDAC cells, *GALNT5* was likely to enhance the NOTCH pathway, thus influencing the immune infiltration in the tumor microenvironment and CAFs to achieve drug resistance.

## Method

### Immunohistochemistry (IHC) of tumor micro assays (TMA)

The tumor micro assays (TMA) contain 150 cases of pancreatic cancer tissues and corresponding adjacent normal tissues. All clinical samples were obtained from Renji Hospital, Shanghai Jiao Tong University School of Medicine, and the study was approved by the Research Ethics Committee of Renji Hospital, Shanghai Jiao Tong University School of Medicine.

IHC was performed under the condition of the EDTA restoration and the dilution ratio of 1:100 of *GALNT5* antibody (poly-antibody, Absin #abs112144). The IHC scores of TMA were the average of scores based on the judgment of two senior pathologists in a blind manner. We defined the score 0 as the “-”, the score 1,2,3 as the “+”, the score 4,5,6 as the “++”, and score 9 as the “+++”. Furthermore, we defined the patients with a score ≤ 4 as the low *GALNT5* expression group and the patients with a score > 4 as the high *GALNT5* expression group.

### Cell culture and reagents

We got human pancreatic cancer cell lines including HPNE, CFPAC-1, Capan-1, MiaPaca-2, PANC1, Patu8988, SW1990, and mouse pancreatic cell line KPC1199 from the Shanghai Cancer Institute, Ren Ji Hospital, School of Medicine, Shanghai Jiao Tong University.

Patu8988, SW1990, MiaPaca-2, PANC1 and KPC1199 were cultured with Dulbecco’s modified Eagle’s medium (DMEM) supplemented with 10% fetal bovine serum (FBS) under the condition of 37 °C and 5% CO2 while Capan-1 was cultured with RPMI medium1640 supplemented with 20% FBS and CFPAC-1 was cultured with IMDM supplemented with 10% FBS.

### Colony-formation assays

We seeded indicated cells in 6-well plates at a density of 2000 cells per well and incubated for 2 weeks. Collected colonies were fixed with 4% paraformaldehyde fix solution and stained with 0.5% (w/v) crystal violet. The shadow areas were calculated by image J.

### Apoptosis assays

To investigate apoptosis, a 488-annexin V/PI cell apoptosis kit (#SB-Y6002, Share-Bio) was used by flow cytometry according to the manufacturer’s instructions.

### Cell cycle assays

Cells are harvested and fixed in cold 70% ethanol for at least 2 h at −20 °C to permeabilize the membranes and preserve cellular structures. After fixation, cells are washed with phosphate-buffered saline (PBS) and treated with RNase A (100 µg/mL) at 37 °C for 30 min to degrade RNA, which could interfere with DNA staining. The cells are then stained with a DNA-binding dye, such as propidium iodide (PI) or 4’,6-diamidino-2-phenylindole (DAPI), at a final concentration of 50 µg/mL, in the presence of 0.1% Triton X-100 to allow dye penetration. Samples are incubated in the dark for 30 min at room temperature. Flow cytometry is performed using a flow cytometer, with data acquisition on the FL2 channel for PI or the appropriate channel for the chosen dye. Data are analyzed using software to determine the distribution of cells across the G0/G1, S, and G2/M phases based on DNA content.

### Cell counting Kit-8

Indicated cells were seeded in 96-well plates at a density of 2000 cells per well. Cell counting kit-8 (CCK8) (#SB-CCK8, Share-Bio) reagent (10 μl/well) mixed with serum-free medium (90 μl/well) was added to each well and incubated for 1 h. A Power Wave XS microplate reader (BIO‐TEK) was used to measure the absorbance at 450 nm.

### IC50 assays

Indicated cells were seeded in 96-well at a density of 3000 cells per well and incubated for three days. The drug concentration gradients in decreasing order were 10 mm, 1 mm, 100 μm, 10 μm, 1 μm, 100 nm, 10 nm, 1 nm, and 0, respectively. Obtained data via CCK8 and calculated cell viability. Plotted the IC50 curve with the cell viability on the vertical axis and the logarithmic concentration on the horizontal axis.

### Immunofluorescence staining

The cells were cultured in chambered coverslips (80826, ibidi) and incubated at a temperature of 37 °C with a condition of 5% CO2 for 48 h. The samples were fixed in 4% paraformaldehyde for a minimum of 15 min, followed by permeabilization with 0.1% Triton X‐100 and shaking for 5–10 min at room temperature. After three washes with PBS for 5 min each, the samples were closed with 2%BSA involved in TBST at RT shaking for 30 min. Incubate samples with specific primary antibodies at 4° overnight and secondary antibodies at room temperature out of light for 1 h. Finally, incubate samples with DAPI for 5 min and capture digital images under Confocal microscopes (Leica, Germany). Primary antibodies include P- γH2A.X(ser139) (1:200, Cell Signaling Technology, #9718). The secondary antibody includes FITC (1:200, Service-bio, GB22303). Orthotopic xenograft tumor slides were performed IF by Service-bio.

### FOI treated assays

FOI is the combination of 5-fluorouracil (10uM), oxaliplatin(8uM), and irinotecan (8uM) in vitro experiments based on IC50 in WT PDAC cells respectively, and Concentrations of 5-fluorouracil (23 mg/kg), oxaliplatin(10 mg/kg), irinotecan(100 mg/kg) for in vivo experiments (i.p) according to manufacturer’s instructions (5-FU, #HY-90006; oxaliplatin, #HY-17371; irinotecan, #HY-16562, MCE).

For IF assays, the “FOI” group was only treated with FOI for 24 h. The “FOI+Si-*MYH9*” group first knocked down *MYH9* by using Si-RNA and was further treated with FOI for 24 h. The “FOI+Si-*MYH9* + VPA” group gave an additional 24 h of Valproic acid (VPA) treatment in addition to the former group. (VPA, 5 mM, #HY-10585, MCE) Colony formation assays were performed under the conditions remaining unchanged except that the FOI and V PA treatments were extended to one week.

### RNA isolation and quantitative RT-PCR

TRI REAGENT (MRC, #TR118) was used to extract total RNA according to the manufacturer’s instructions. cDNAs were synthesized using the PrimeScript™ RT Master Mix (Takara, #RR036A) based on the manufacturer’s introduction. Quantitative real‐time PCR was performed with FastStart Universal SYBR Green Master (Roche, 04913914001) on a 7500 Real-time PCR system (Applied Biosystems) at the recommended thermal cycling settings: one initial cycle at 95 °C for 10 min followed by 40 cycles of 15 s at 95 °C and 60 s at 60 °C. Relative mRNA expression was calculated by the 2^−ΔΔCt^ method and normalized to *18S* mRNA levels. Primer sequences are listed in Supplementary Table 1.

### Western blotting

The proteins were extracted, followed by the lysis of cells or tissues with the mix of WB IP lysis and extraction buffer (#89900, Thermo-Fisher) and proteinase phosphatase inhibitors (#P002, NCM Biotech). The supernatant was obtained after centrifugation at 12000 rpm at 4 °C and then, mixed with one-fifth of sodium dodecyl sulfate (SDS) - loading buffer with a final total volume and boiled for 15 min. The same total amount of proteins was separated by SDS-PAGE (polyacrylamide gel electrophoresis) at 80 V for 35 min and 120 V for 70 min and then transfected to the nitrocellulose filter membrane (NC) for 17 min. Afterward, the membranes were washed using TBST (50 mM TRIS + 150 mM sodium chloride + 0.1% Tween 20, pH 7.4) and blocked by adding a 5% non-fat milk solution in TBST for at least 1 h at room temperature.

Subsequently, wash membranes with TBST three times for 5 min each time and incubate with specific primary antibodies at 4°C overnight. Wash membranes again with TBST three times for 10 min each time and incubate with the corresponding secondary antibodies. Finally, the membranes were detected by the chemiluminescence system (BIO-RAD).

Primary antibodies include *GALNT5* (1:1000, Absin #abs112144), the DNA damage antibody sampler kit (Cell Signaling Technology #9947) (1. 1:1000, Phospho-ATM (Ser1981) #5883, 2. 1:1000, Phospho-Chk2 (Thr68) #2197, 3. 1:1000, Phospho-ATR (Ser428) #2853, 4. 1:1000, Phospho-Chk1 (Ser345) #2348), Notch1(NICD) (1:1000, Cell Signaling Technology antibody, #3608), Jagged1 (1:1000, Cell Signaling Technology antibody #70109), *MYH9* (1:1000, Proteintech # 81204-1-RR) and Beta- Actin (1:1000, Cell Signaling Technology #4970) Secondary antibodies included goat anti-rabbit IgG, and HRP-linked antibodies (1:10000, #7074, Cell Signaling Technology).

### gene set enrichment analysis (GSEA)

Gene set enrichment analysis (GSEA) was performed among the samples of TCGA datasets and GEO datasets GSE16515. We divided all samples into two groups based on *GALNT5* expression and adopted “h.all.v2023.1.Hs.symbols.gmt” and “c2.cp.kegg.v2023.1.Hs.symbols.gmt” two gene sets databases to seek shared pathways. │NES│ ≥ 1, NOM p < 0.05, FDR < 0.25.

### Co-immunoprecipitation (Co-IP)

Lysis cells using the WB IP lysis and extraction buffer (#89900, Thermo-Fisher) supplemented with proteinase phosphatase inhibitors (#P002, NCM Biotech). The supernatant was obtained after centrifugation at 12000 rpm at 4 °C. Incubate beads with antibodies for 30 min first and then incubate the samples for 1 h at room temperature. Subsequently, perform western blots with specific antibodies.

### Knockdown and overexpression assay

Knockdown and overexpression sequences targeting *GALNT5* and knockdown sequences targeting *MYH9* are listed in Supplementary Table 1.

### Animal model studies

The mice experiments were approved by the Renji Hospital Animal Care and Use Committee. Mice were manipulated and housed according to the criteria outlined in the Guide for the Care and Use of Laboratory Animals prepared by the National Academy of Sciences and published by the NIH (Bethesda, MD).

### Orthotopic xenograft model

The male C57BL/6 mice were purchased from the Leagene company, which were 6–8 weeks old with a weight between 20 and 25 g. All mice were randomly divided into 4 groups which were Ctrl-vehicle, Ctrl-FOI-treated, Sh*GALNT5*-Vehicle, and Sh*GALNT5*-FOI-treated groups respectively. Indicated 1 × 10^6^ luciferase‐expressing KPC1199 cells suspended in 25 μl DMEM were transplanted into the body of the pancreas. Postinoculation, mice were treated with intraperitoneal administration of FOI [oxaliplatin (10 mg/kg), 5-fluorouracil (23 mg/kg), and irinotecan (100 mg/kg)] or vehicle (DMSO) alone once a week for four weeks. The luciferase signal intensity was examined using the IVIS spectrum (Calliper Life Sciences) after intraperitoneal injection of D‐luciferin (#40901ES03, YEASEN) into the mice once a week. Finally, five mice were randomly selected from each of the four groups for bioluminescent imaging whose emission was measured by Living Image software, version 4.5.3. The pancreas with orthotopic xenograft tumor were removed, weighed, and paraffin-embedded four weeks post-inoculation.

PDX model tumor samples are first obtained from patients diagnosed with pancreatic ductal adenocarcinoma (PDAC) during surgical resection or biopsy. The samples are then cut into small fragments (2 × 2 × 2) mm³ under sterile conditions. These fragments are implanted subcutaneously into the flanks of NOD/SCID using a trocar. The mice are monitored regularly for tumor growth, and the tumors are measured using calipers. Once tumors reach a size of 1.5 cm³, they are harvested, and the process can be repeated for expansion or downstream analysis.

### Statistical analysis

SPSS 26.0 software and GraphPad Prism 8 software were adopted. we performed two-tailed unpaired student’s t-test to compare two experimental groups and two-way ANOVA was adopted to compare three or more experimental groups. The error bars in the figures represent the mean ± standard deviation (SD). Significant difference was defined as *p < 0.05, **p < 0.01, ***p < 0.001, ns (no significance) *p* > 0.05. All results were repeated at least three independent times.

## Supplementary information

supplementary figures

Supplementary table3-4

Supplementary table1-2

Review WB raw data

P value

## Data Availability

The datasets generated and/or analyzed during the current study are available from the corresponding author on reasonable request
