# Supplementary material for: Oncogenic GALNT5 confers FOLFIRINOX resistance via activating the MYH9/ NOTCH/ DDR axis in pancreatic ductal adenocarcinoma
Source: Cell Death Dis. 2024 Oct 21;15(10):767. doi: 10.1038/s41419-024-07110-w (PMC11493973; doi:10.1038/s41419-024-07110-w)
Supplement: Supplementary file 1 — supplementary figures [file 41419_2024_7110_MOESM1_ESM.pdf]

Figure S1 clinical significance analysis of GALNT5 in TCGA database

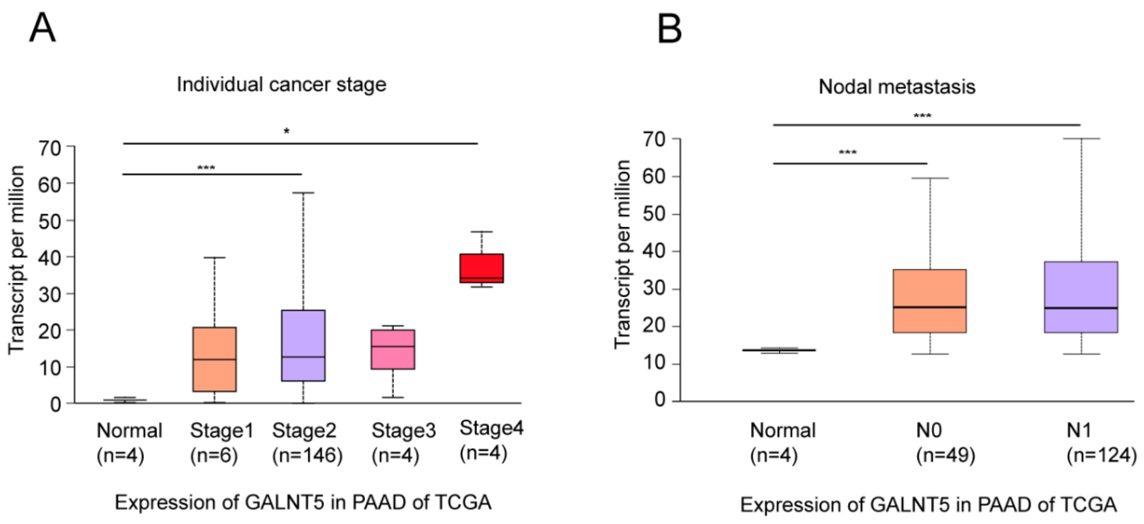

(A) TCGA database demonstrating the expressions of GALNT5 in different individual cancer stage (Normal, n=4; stage1, n=6; stage2, n=146; stage3, n=4; stage4, n=4) of PDAC (Two-tailed unpaired Student t-test, ***$p<0.001$)

(B) TCGA database showing the relationship of GALNT5 with nodal metastasis in PDAC (Normal, n=4; N0, n=49; N1, n=124, Two-tailed unpaired Student t-test, ***$p<0.001$)

Figure S2 Detection of the expression level of GALNT5 in pancreatic cancer cell lines and verification of the construction efficiency of stable metastatic strains

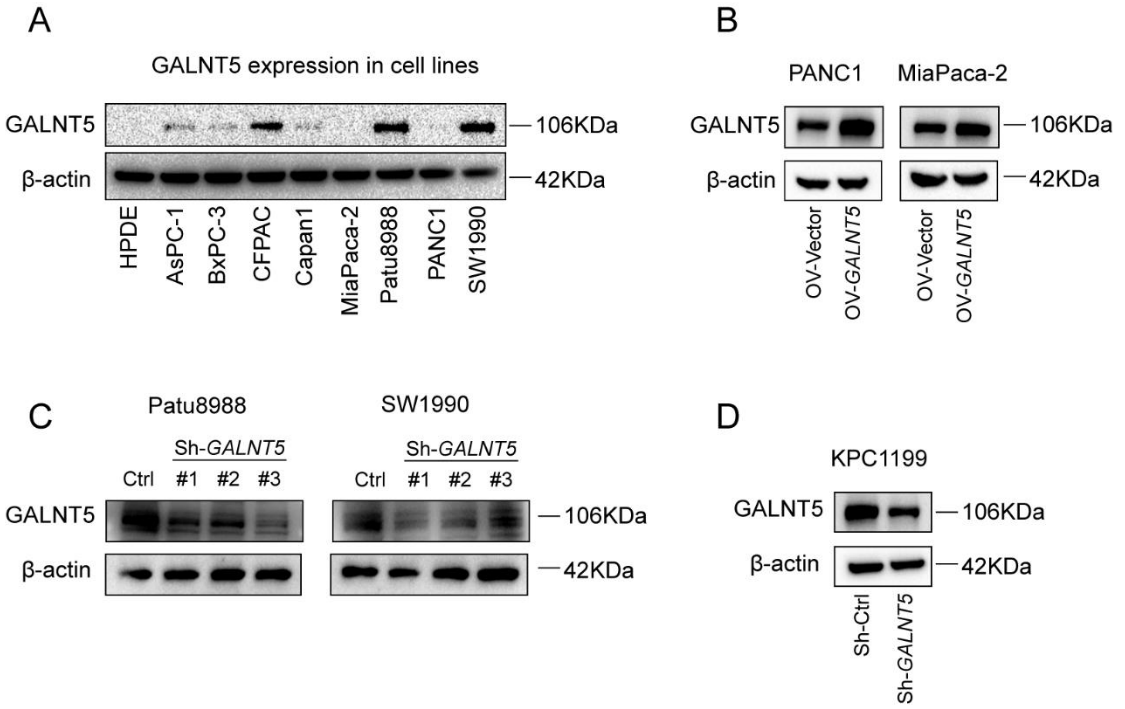

(A) Detection of the expression level of GALNT5 in pancreatic cancer cell lines

(B-D) Verification of construction efficiency of GALNT5 overexpressed stable transmutation (B, D) strain and knockdown stable transmutation strain (C)

Figure S3 Alterations of 5-FU IC50 in pancreatic cancer cells after knockdown and overexpression of GALNT5

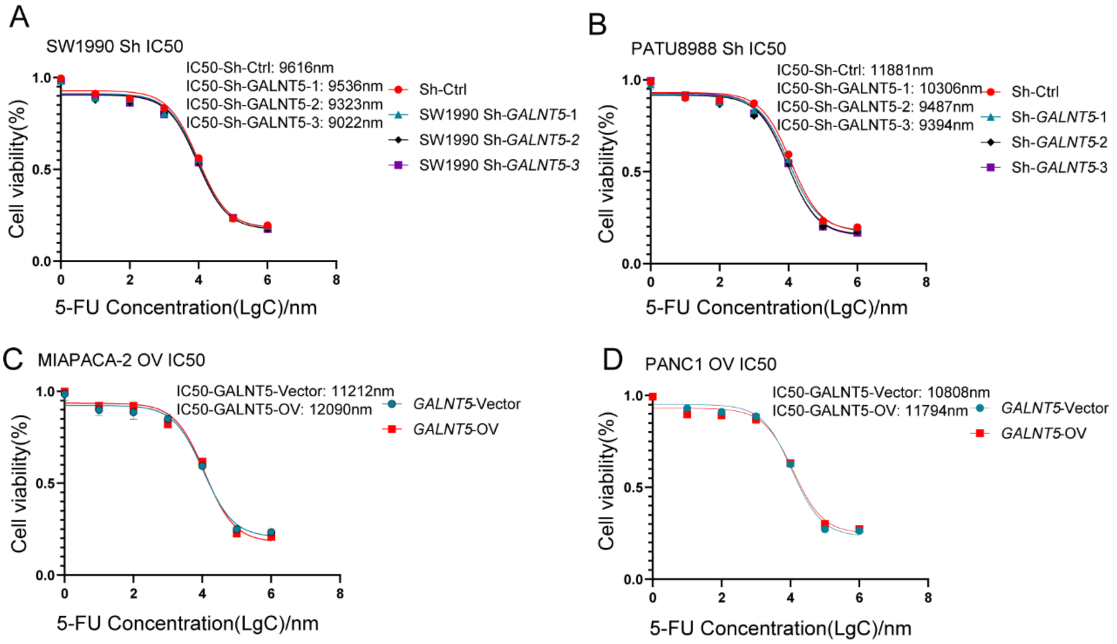

(A-B) Changes of 5-FU IC50 in pancreatic cancer cells after knocking down GALNT5

(C-D) Changes of 5-FU IC50 in pancreatic cancer cells after overexpressing GALNT5

Figure S4 GSEA analysis based on GALNT5 expression

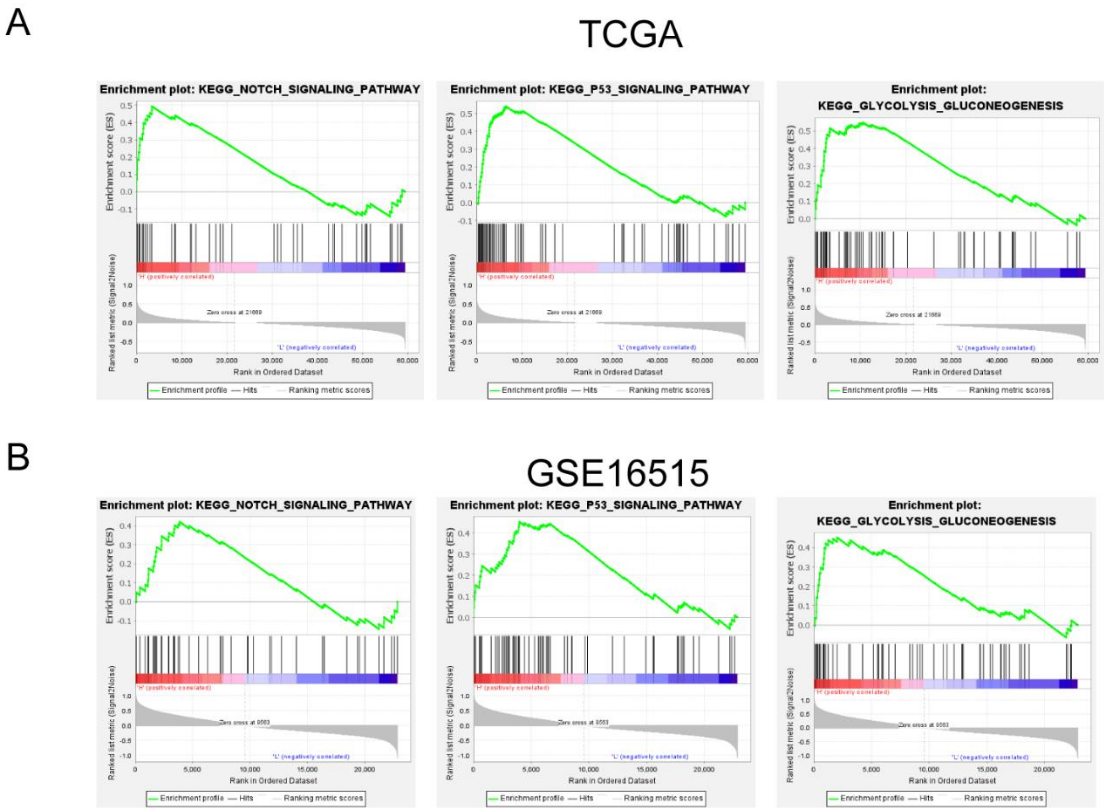

(A-B) GSEA was performed on TCGA datasets (A) and GEO datasets GSE16515 (B) based on GALNT5 expression.
