# Supplementary material for: Oncogenic GALNT5 confers FOLFIRINOX resistance via activating the MYH9/ NOTCH/ DDR axis in pancreatic ductal adenocarcinoma
Source: Cell Death Dis. 2024 Oct 21;15(10):767. doi: 10.1038/s41419-024-07110-w (PMC11493973; doi:10.1038/s41419-024-07110-w)
Supplement: Supplementary file 3 — Supplementary table1-2 [file 41419_2024_7110_MOESM3_ESM.docx]

**Supplementary Table 1**

**. Primers used in PCR.**

| **Primer Name** | **Forward Primer (+)**  **Reverse Primer (-)** | **Sequence** |
| --- | --- | --- |
| NOTCH1 | **+** | 5’-GAGGCGTGGCAGACTATGC-3’ |
|  | **-** | 5’-CTTGTACTCCGTCAGCGTGA-3’ |
| JAG1 | **+** | 5’- GTCCATGCAGAACGTGAACG -3’ |
|  | **-** | 5’- GCGGGACTGATACTCCTTGA -3’ |
| HES1 | **+** | 5’-TCAACACGACACCGGATAAAC-3’ |
|  | **-** | 5’-GCCGCGAGCTATCTTTCTTCA-3’ |
| HEY1 | **+** | 5’-GTTCGGCTCTAGGTTCCATGT-3’ |
|  | **-** | 5’-CGTCGGCGCTTCTCAATTATTC-3’ |
| CCND1 | + | 5’- GCTGCGAAGTGGAAACCATC -3’ |
|  | - | 5’- CCTCCTTCTGCACACATTTGAA -3’ |
| C-Myc | + | 5’-GGCTCCTGGCAAAAGGTCA-3’ |
|  | - | 5’-CTGCGTAGTTGTGCTGATGT-3’ |
| GAPDH | + | 5’-GGAGCGAGATCCCTCCAAAAT-3’ |
|  | - | 5’-GGCTGTTGTCATACTTCTCATGG-3’ |
| LDHA | + | 5’-ATGGCAACTCTAAAGGATCAGC-3’ |
|  | - | 5’-CCAACCCCAACAACTGTAATCT-3’ |
| PKM2 | + | 5’-ATGTCGAAGCCCCATAGTGAA-3’ |
|  | - | 5’-TGGGTGGTGAATCAATGTCCA-3’ |
| PFKP | + | 5’-GCATGGGTATCTACGTGGGG-3’ |
|  | - | 5’-CTCTGCGATGTTTGAGCCTC-3’ |
| GLUT | + | 5’-GGCCAAGAGTGTGCTAAAGAA-3’ |
|  | - | 5’-ACAGCGTTGATGCCAGACAG-3’ |
| HKII | + | 5’-GAGCCACCACTCACCCTACT-3’ |
|  | - | 5’-CCAGGCATTCGGCAATGTG-3’ |
| P53 | + | 5’-CAGCACATGACGGAGGTTGT-3’ |
|  | - | 5’-TCATCCAAATACTCCACACGC-3’ |
| PUMA | + | 5’-GACCTCAACGCACAGTACGAG-3’ |
|  | - | 5’-AGGAGTCCCATGATGAGATTGT-3’ |
| NOXA | + | 5’-ACCAAGCCGGATTTGCGATT-3’ |
|  | - | 5’-ACTTGCACTTGTTCCTCGTGG-3’ |
| FAS | + | 5’-TCTGGTTCTTACGTCTGTTGC-3’ |
|  | - | 5’-CTGTGCAGTCCCTAGCTTTCC-3’ |
| CASP3 | + | 5’-AGAGGGGATCGTTGTAGAAGTC-3’ |
|  | **-** | 5’-ACAGTCCAGTTCTGTACCACG-3’ |
| BAX | **+** | 5’-CCCGAGAGGTCTTTTTCCGAG-3’ |
|  | **-** | 5’-CCAGCCCATGATGGTTCTGAT-3’ |
| BAK | **+** | 5’-GTTTTCCGCAGCTACGTTTTT-3’ |
|  | **-** | 5’-GCAGAGGTAAGGTGACCATCTC-3’ |
| 18S | **+** | 5’-GGCCCTGTAATTGGAATGAGTC-3’ |
|  | **-** | 5’-CCAAGATCCAACTACGAGCTT-3’ |

**Supplementary Table 2**

**GALNT5 KD OV sequence**

GALNT5 knockdown sequence (Human):

ShGALNT5-1:

F: 5’-GGCCUGUCCAGUAAUCGAA (dT)(dT)-3’

R: 5’-UUCGAUUACUGGACAGGCC (dT)(dT)-3’

ShGALNT5-2

F: 5’-GUUACAUGACAGUGGAUAA (dT)(dT)-3’

R: 5’-UUAUCCACUGUCAUGUAAC (dT)(dT)-3’

ShGALNT5-3

F: 5’-GAGCUAGUGGUGUGCUUAU (dT)(dT)-3’

R: 5’-AUAAGCACACCACUAGCUC (dT)(dT)-3’

Galnt5 knockdown sequence (Mouse)

ShGalnt5-1

F: 5’- CAGCGAUACAUCAAAGCAA (dT)(dT)-3’

R: 5’- UUGCUUUGAUGUAUCGCUG (dT)(dT)-3’

ShGalnt5-2

F: 5’- CCGUAUGAAGACAGUGGAA (dT)(dT)-3’

R: 5’- UUCCACUGUCUUCAUACGG (dT)(dT)-3’

ShGalnt5-3

F: 5’- GGGACUCCAUACAGCAAGA (dT)(dT)-3’

R: 5’- UCUUGCUGUAUGGAGUCCC (dT)(dT)-3’

GALNT5 overexpression sequence

gaattcATGGCCTCCGGTGCGTATAACCCGTATATAGAGATAATTGAACAACCCAGGCAGAGGGGAATGCGTTTTAGATACAAATGTGAAGGGCGATCAGCAGGCAGCATTCCAGGGGAGCACAGCACAGACAACAACCGAACATACCCTTCTATCCAGATTATGAACTATTATGGAAAAGGAAAAGTGAGAATTACATTAGTAACAAAGAATGACCCATATAAACCTCATCCTCATGATTTAGTTGGAAAAGACTGCAGAGACGGCTACTATGAAGCAGAATTTGGACAAGAACGCAGACCTTTGTTTTTCCAAAATTTGGGTATTCGATGTGTGAAGAAAAAAGAAGTAAAAGAAGCTATTATTACAAGAATAAAGGCAGGAATCAATCCATTCAATGTCCCTGAAAAACAGCTGAATGATATTGAAGATTGTGACCTCAATGTGGTGAGACTGTGTTTTCAAGTTTTTCTCCCTGATGAACATGGTAATTTGACGACTGCTCTTCCTCCTGTTGTCTCGAACCCAATTTATGACAACCGTGCTCCAAATACTGCAGAATTAAGGATTTGTCGTGTAAACAAGAATTGTGGAAGTGTCAGAGGAGGAGATGAAATATTTCTACTTTGTGACAAAGTTCAGAAAGATGACATAGAAGTTCGTTTTGTGTTGAACGATTGGGAAGCAAAAGGCATCTTTTCACAAGCTGATGTACACCGTCAAGTAGCCATTGTTTTCAAAACTCCACCATATTGCAAAGCTATCACAGAACCCGTAACAGTAAAAATGCAGTTGCGGAGACCTTCTGACCAGGAAGTTAGTGAATCTATGGATTTTAGATATCTGCCAGATGAAAAAGATACTTACGGCAATAAAGCAAAGAAACAAAAGACAACTCTGCTTTTCCAGAAACTGTGCCAGGATCACGTAGAAACAGGGTTTCGCCATGTTGACCAGGATGGTCTTGAACTCCTGACATCAGGTGATCCACCCACCTTGGCCTCCCAAAGTGCTGGGATTACAGTTAATTTTCCTGAGAGACCAAGACCTGGTCTCCTCGGTTCAATTGGAGAAGGAAGATACTTCAAAAAAGAACCAAACTTGTTTTCTCATGATGCAGTTGTGAGAGAAATGCCTACAGGGGTTTCAAGTCAAGCAGAATCCTACTATCCCTCACCTGGGCCCATCTCAAGTGGATTGTCACATCATGCCTCAATGGCACCTCTGCCTTCTTCAAGCTGGTCATCAGTGGCCCACCCCACCCCACGCTCAGGCAATACAAACCCACTGAGTAGTTTTTCAACAAGGACACTTCCTTCTAATTCGCAAGGTATCCCACCATTCCTGAGAATACCTGTTGGGAATGATTTAAATGCTTCTAATGCTTGCATTTACAACAATGCCGATGACATAGTCGGAATGGAAGCGTCATCCATGCCATCAGCAGATTTATATGGTATTTCTGATCCCAACATGCTGTCTAATTGTTCTGTGAATATGATGACAACCAGCAGTGACAGCATGGGAGAGACTGATAATCCAAGACTTCTGAGCATGAATCTTGAAAACCCCTCATGTAATTCAGTGTTAGACCCAAGAGACTTGAGACAGCTCCATCAGATGTCCTCTTCCAGTATGTCAGCAGGCGCCAATTCCAATACTACTGTTTTTGTTTCACAATCAGATGCATTTGAGGGATCTGACTTCAGTTGTGCAGATAACAGCATGATAAATGAGTCGGGACCATCAAACAGTACTAATCCAAACAGTCATGGTTTTGTTCAAGATAGTCAGTATTCAGGTATTGGCAGTATGCAAAATGAGCAATTGAGTGACTCCTTTCCATATGAATTTTTTCAAGTAGATTACAAGGATGACGACGATAAGTAAggatcc

MYH9 knockdown sequence (Human)

SiMYH9-1:

F: 5’- GGGUAUCAAUGUGACCGAU (dT)(dT)-3’

R: 5’- AUCGGUCACAUUGAUACCC (dT)(dT)-3’

SiMYH9-2:

F: 5’- CCACCAACCUCACAGAAGA (dT)(dT)-3’

R: 5’- UCUUCUGUGAGGUUGGUGG (dT)(dT)-3’

SiMYH9-3

F: 5’- CGGCAAGGUGGAUUACAAA (dT)(dT)-3’

R: 5’- UUUGUAAUCCACCUUGCCG (dT)(dT)-3’
