# Supplementary material for: Oncogenic GALNT5 confers FOLFIRINOX resistance via activating the MYH9/ NOTCH/ DDR axis in pancreatic ductal adenocarcinoma
Source: Cell Death Dis. 2024 Oct 21;15(10):767. doi: 10.1038/s41419-024-07110-w (PMC11493973; doi:10.1038/s41419-024-07110-w)

Figure2 p-value

(data from “The University of Alabama at Birmingham Cancer data analysis Portal”)

2A

Normal-vs-Grade 1 p=1.178020E-04

Normal-vs-Grade 2 p<1E-12

Normal-vs-Grade 3 p=1.39140032828777E-10

Grade 1-vs-Grade 2 p=1.599790E-02

Grade 1-vs-Grade 3 p=2.476100E-03

Grade 1-vs-Grade 4 p=1.178020E-04

Grade 2-vs-Grade 4 p<1E-12

Grade 3-vs-Grade 4 p=1.39140032828777E-10

2B

Normal-vs-TP53-Mutant p=1.56450999999613E-05

TP53-Mutant-vs-TP53-NonMutant p=1.8703999999925E-05

Figure3 p-value


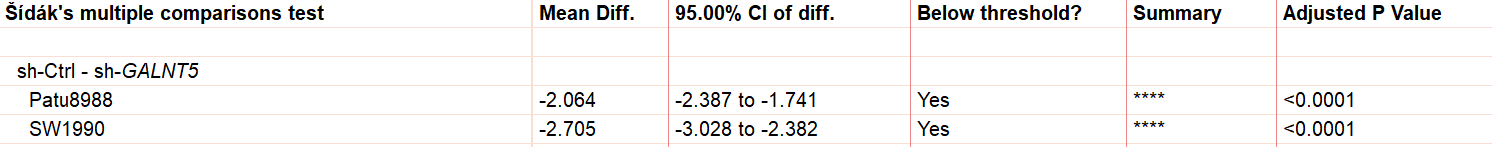
3C


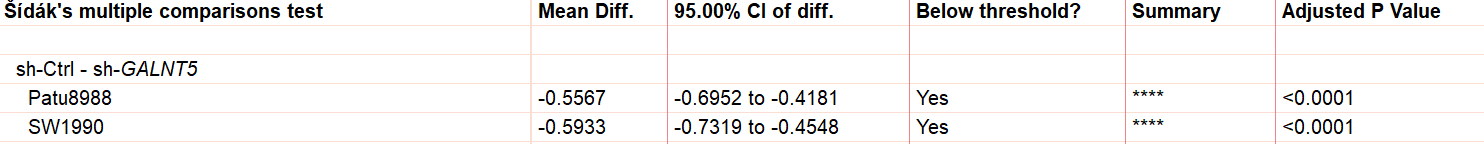
3D


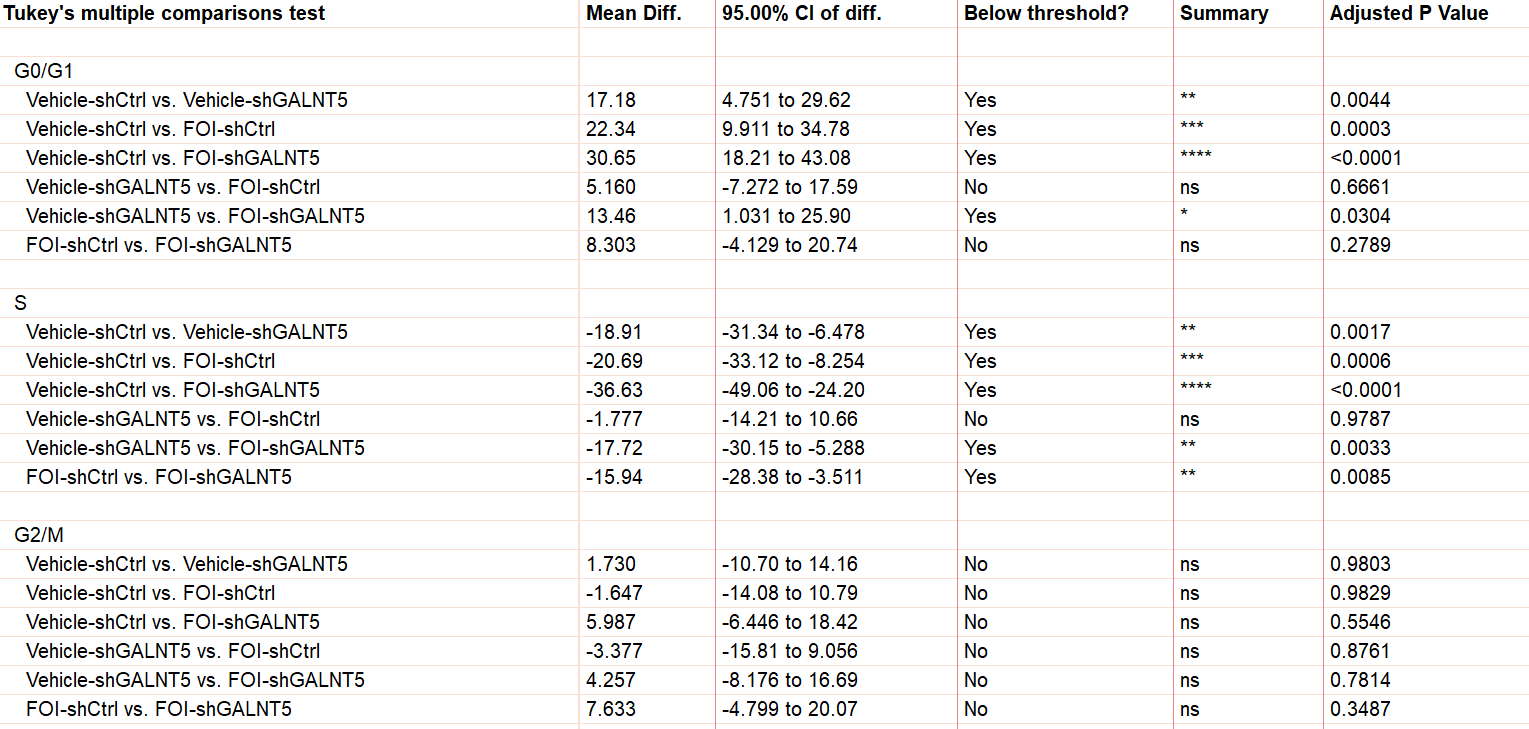
3E


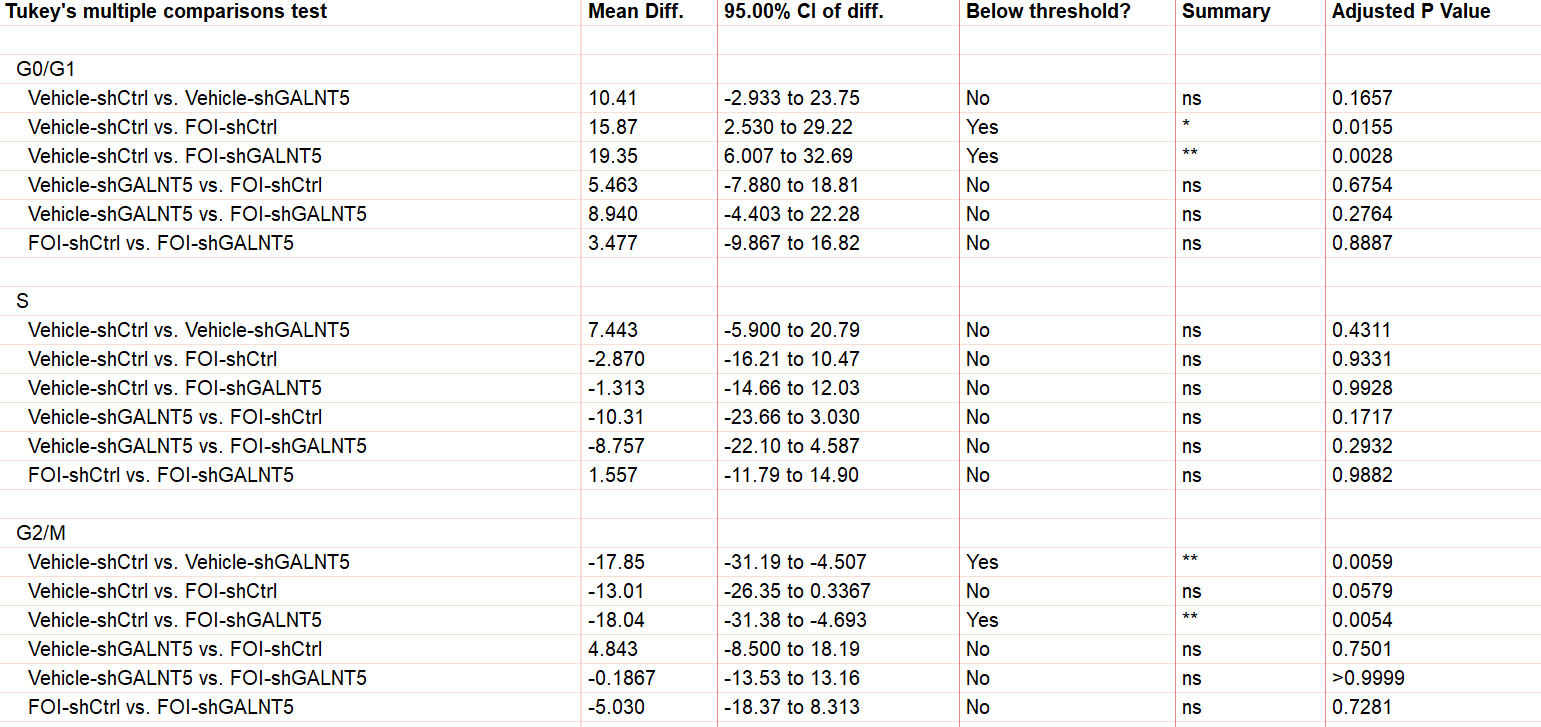
3F


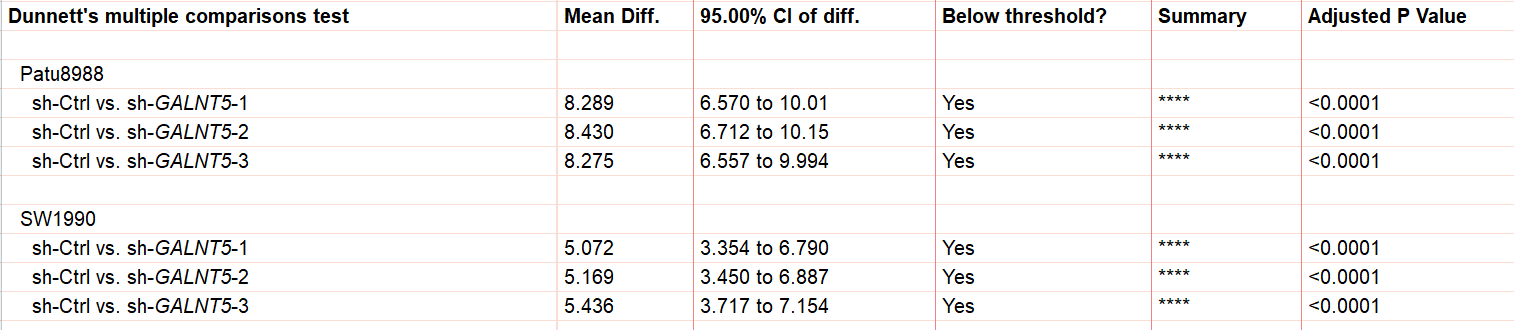
3O


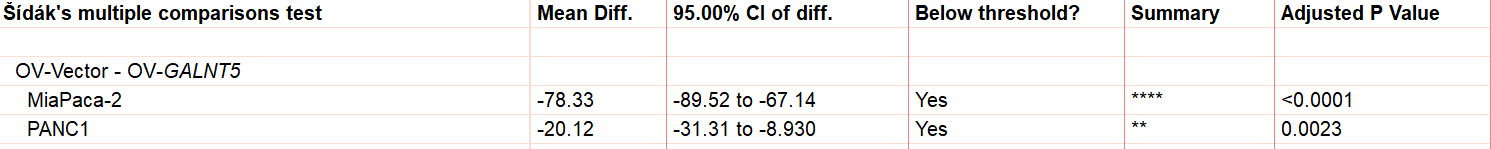
3P


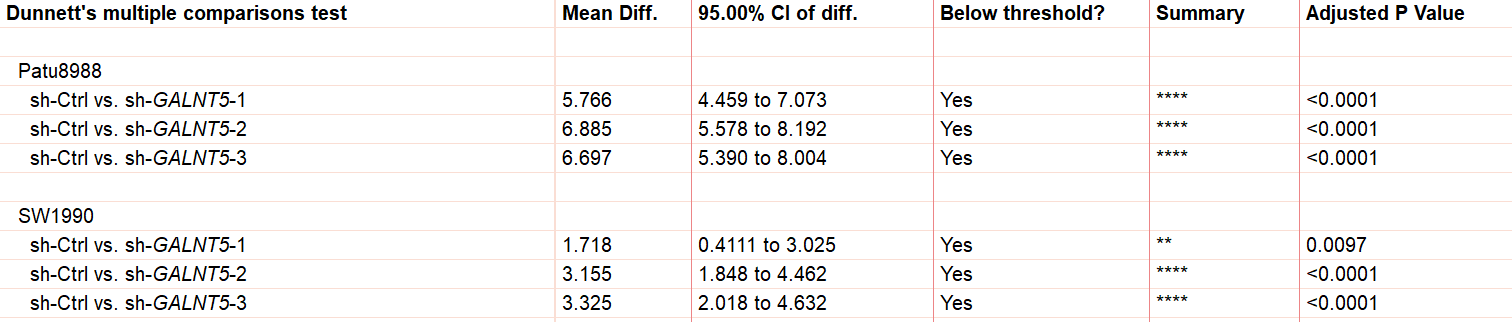
3Q


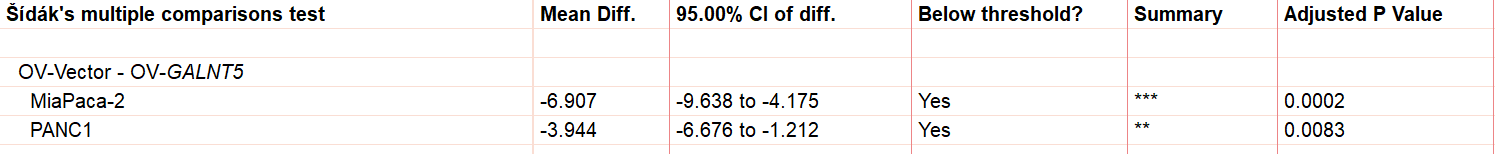
3R


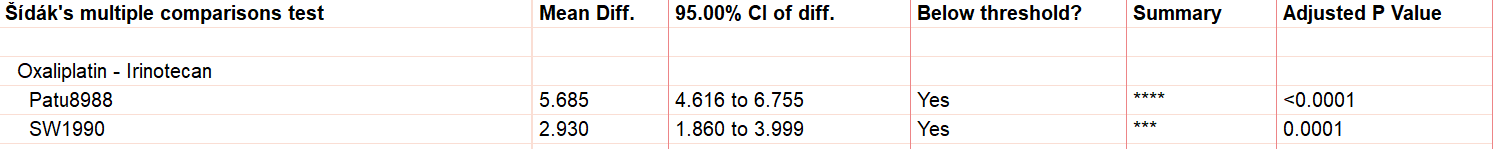
3S


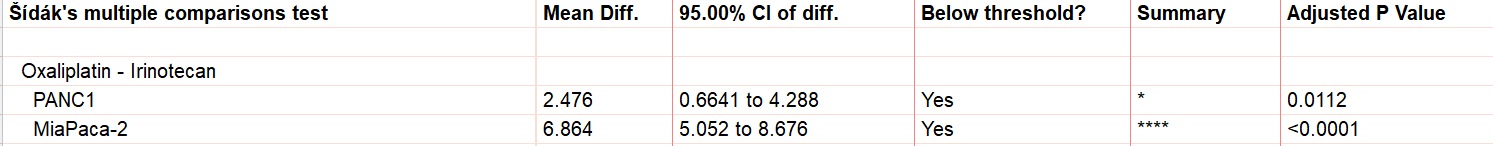
3T

Figure4 p-value


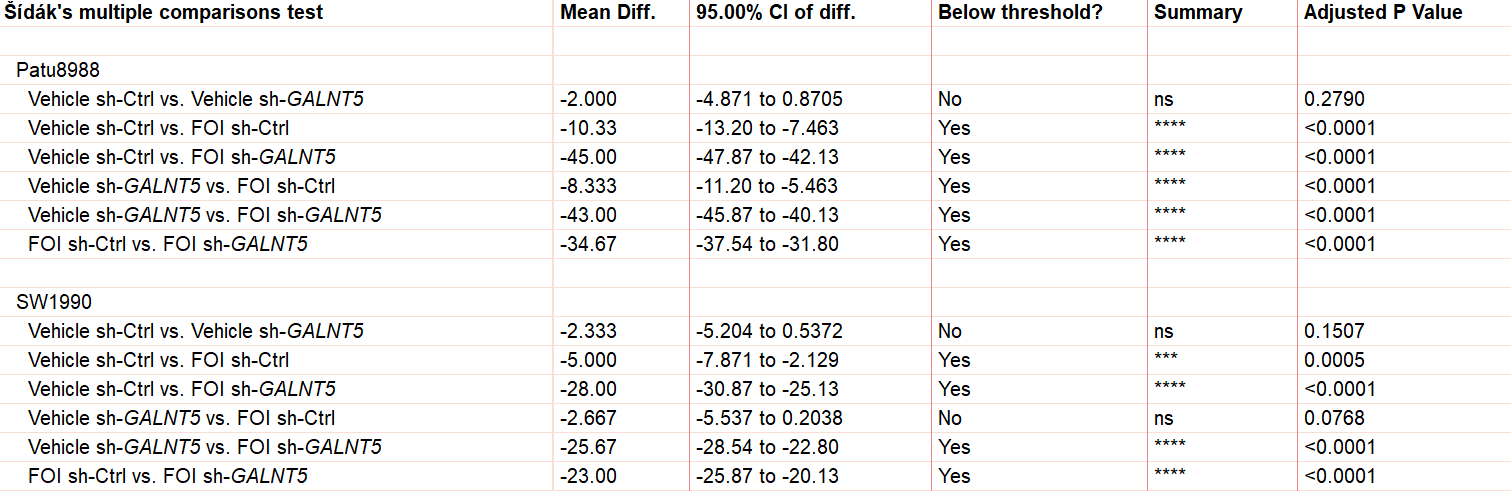
4I


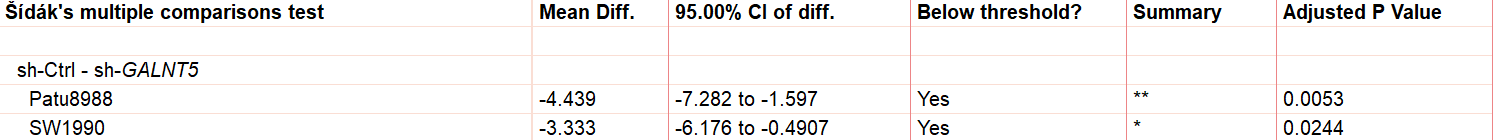
4J


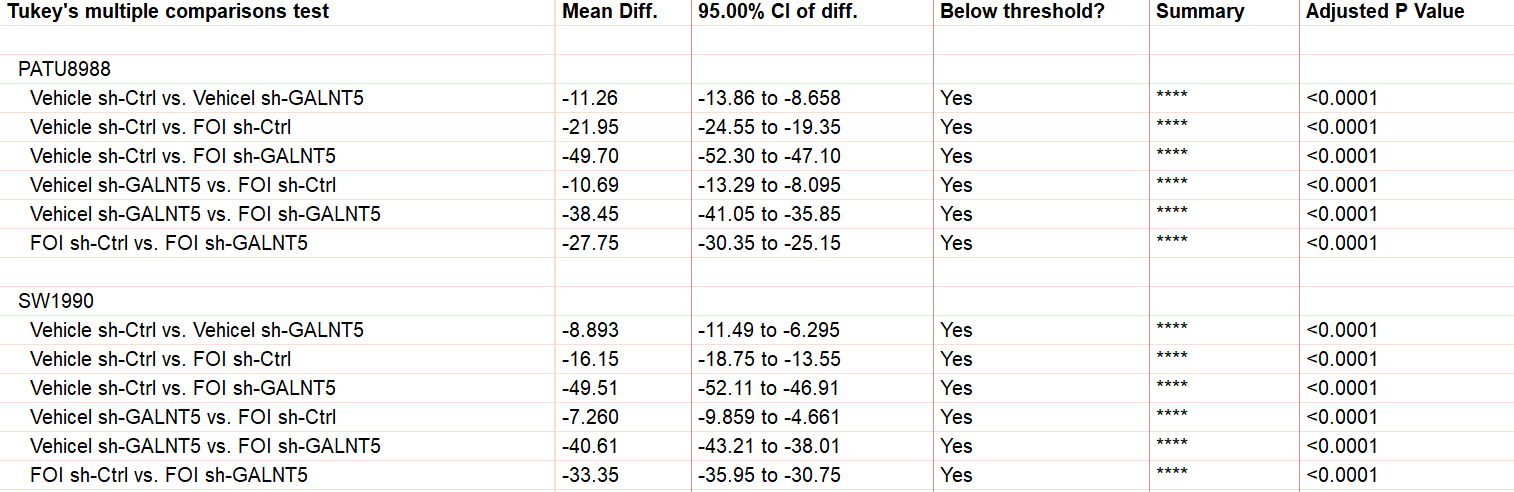
4M


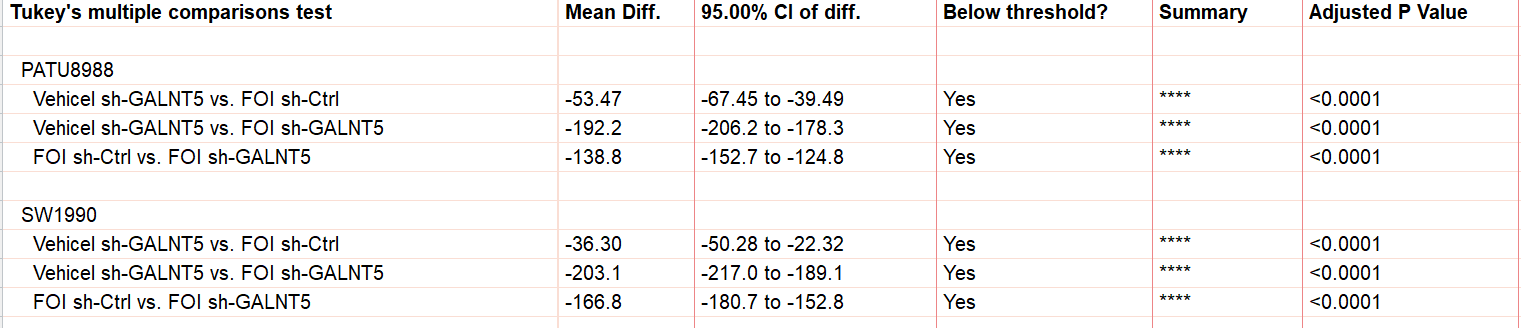
4N

Figure5 p-value

5B


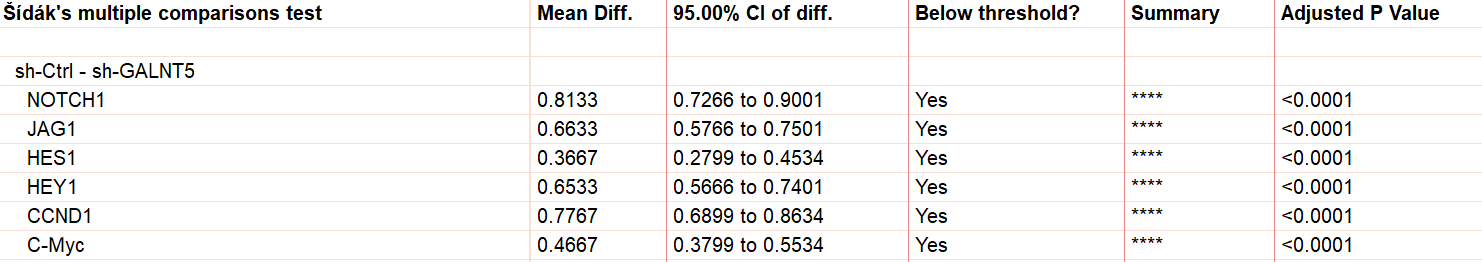
NOTCH pathway


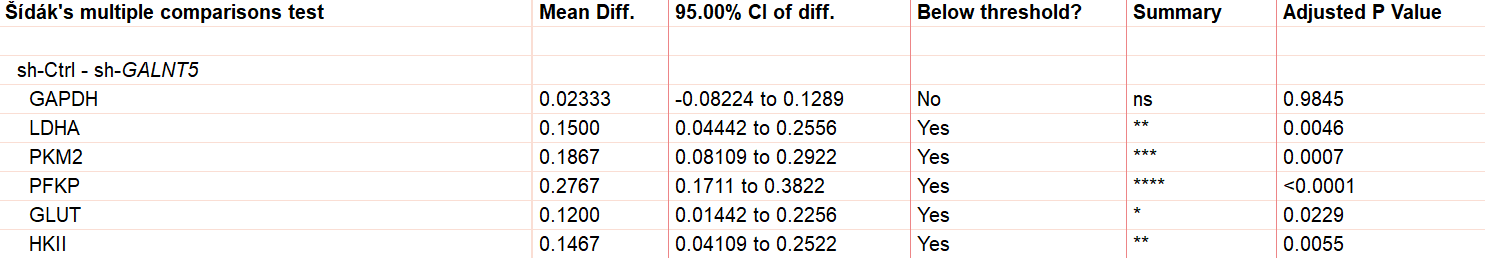
Glycolysis pathway


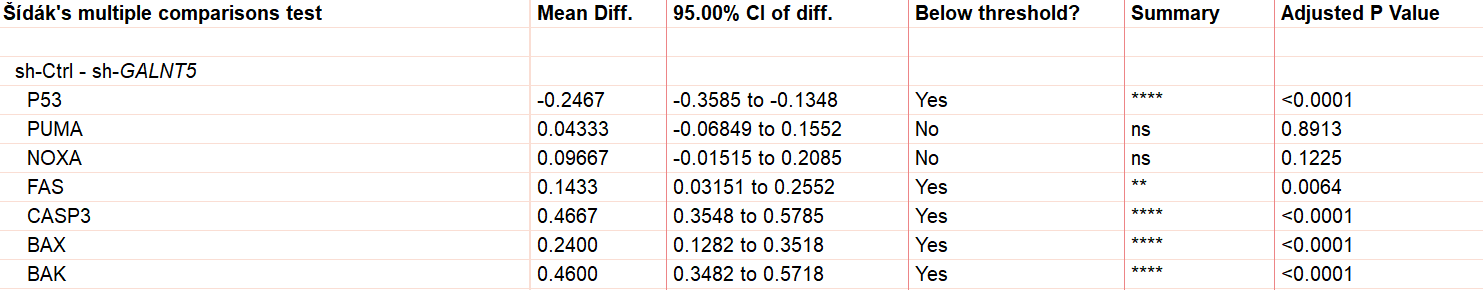
P53 pathway


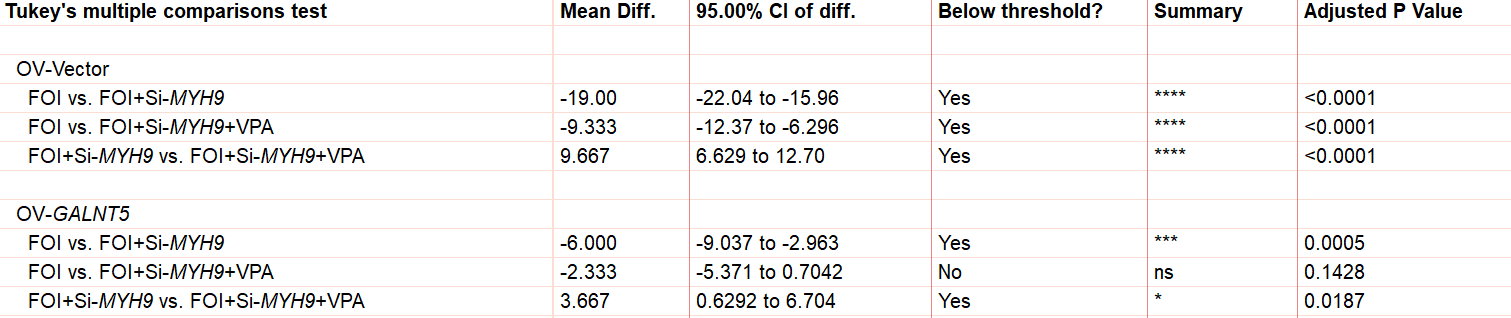
5H


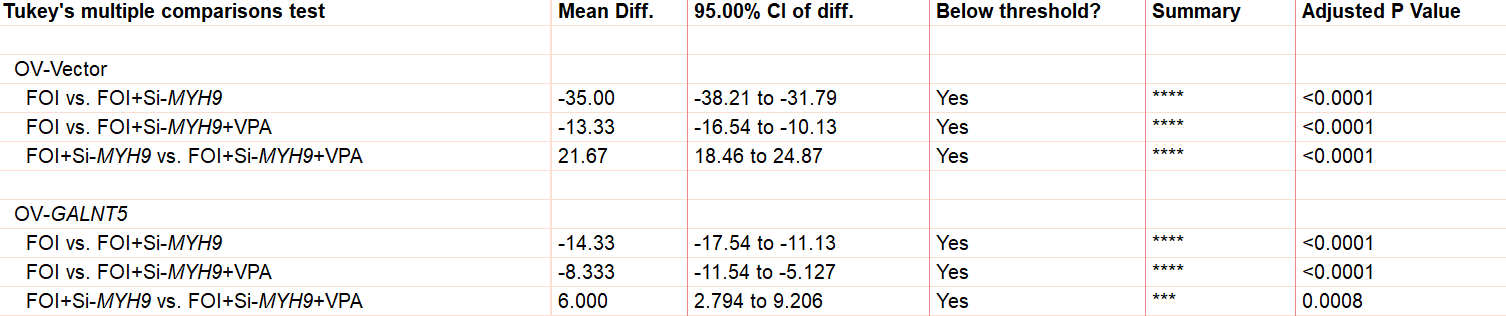
5J


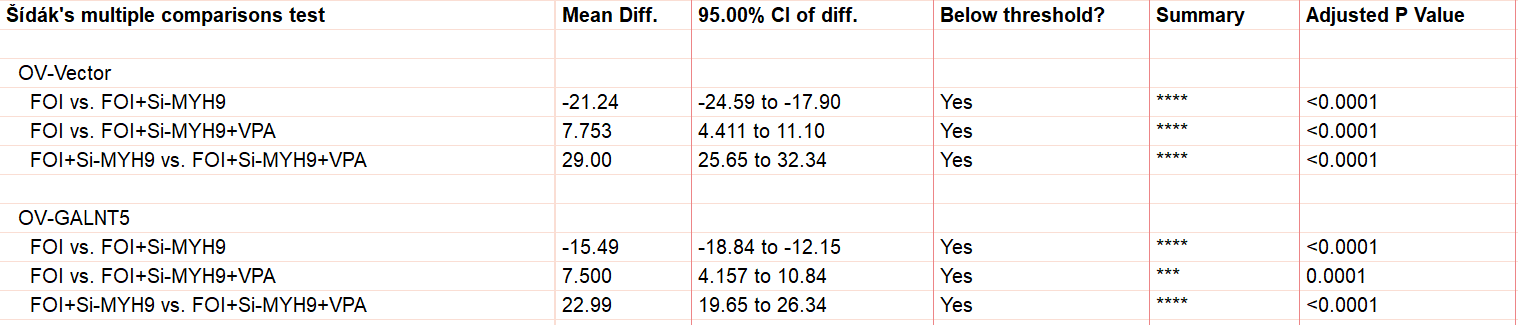
5L


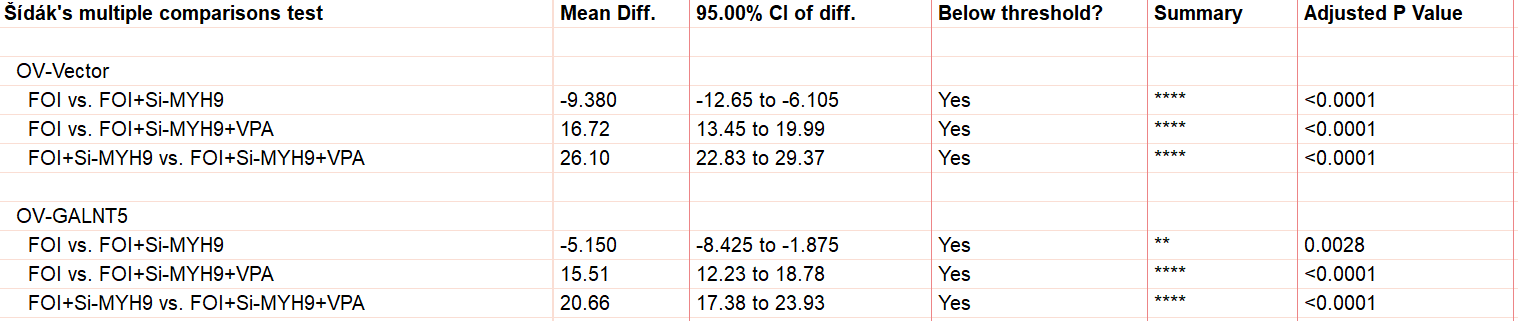
5N


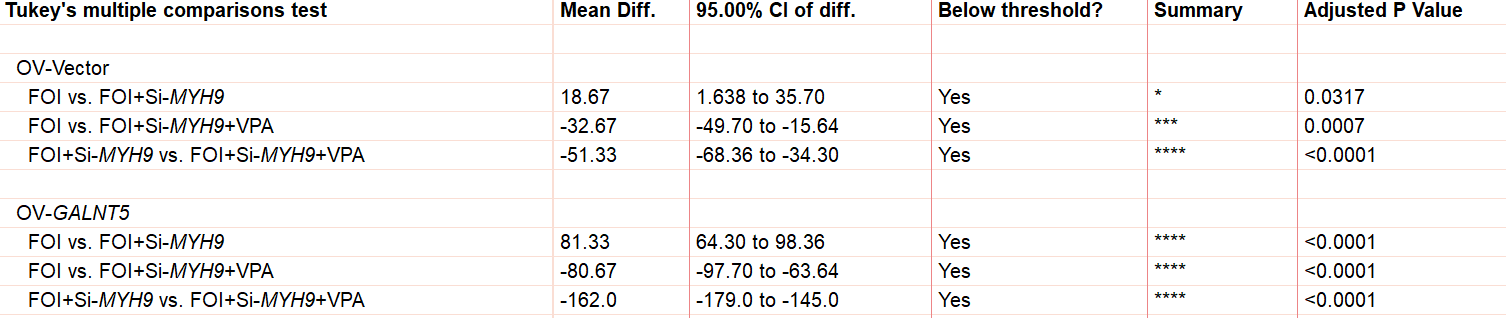
5P


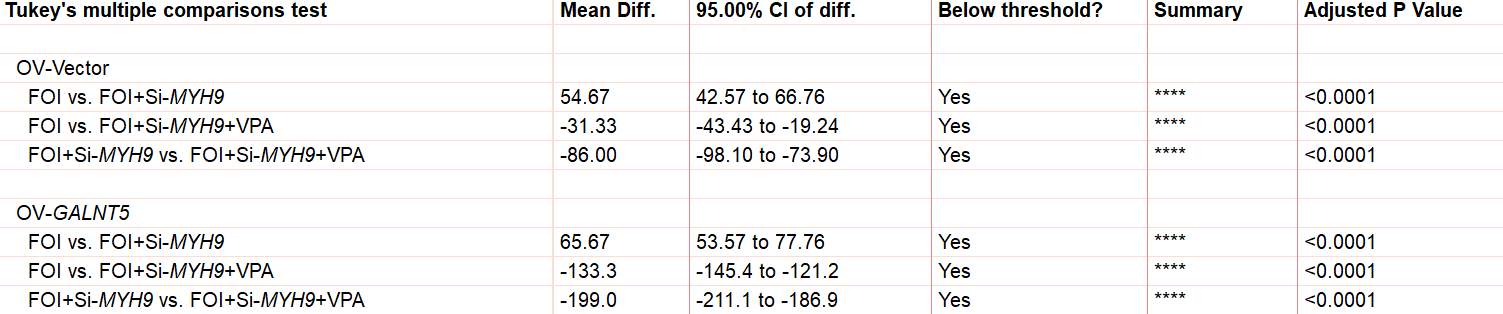
5R

Figure6 p-value


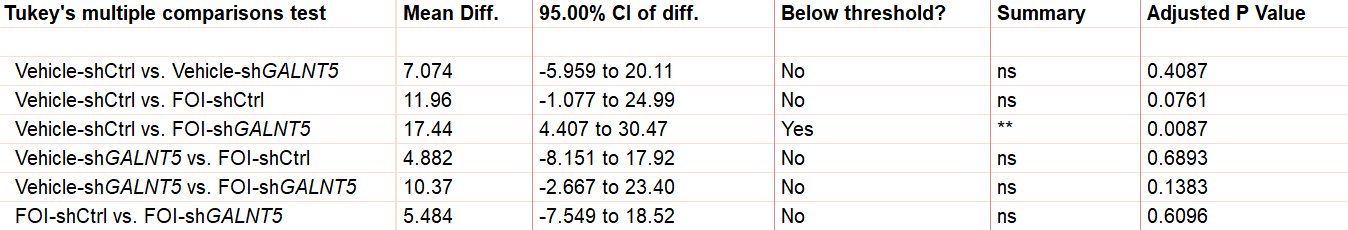
6B


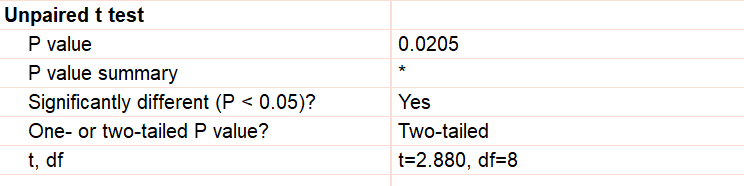
6C


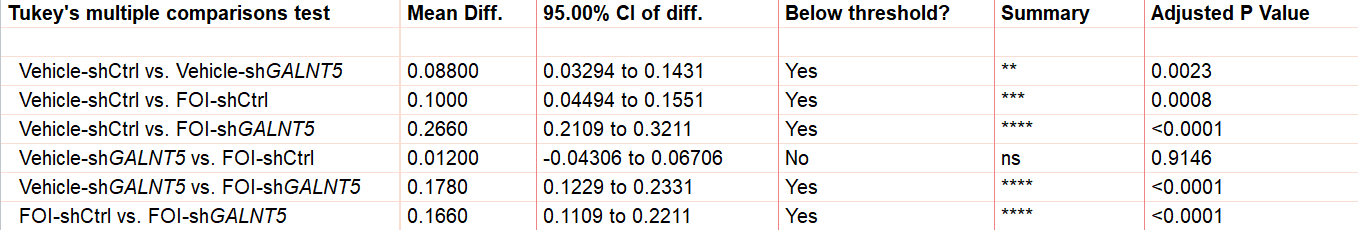
6E


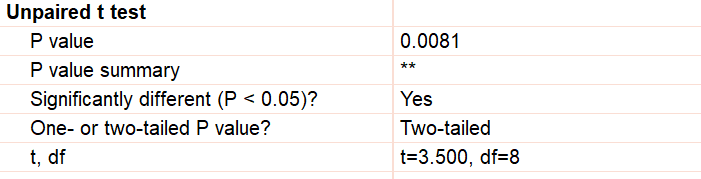
6F


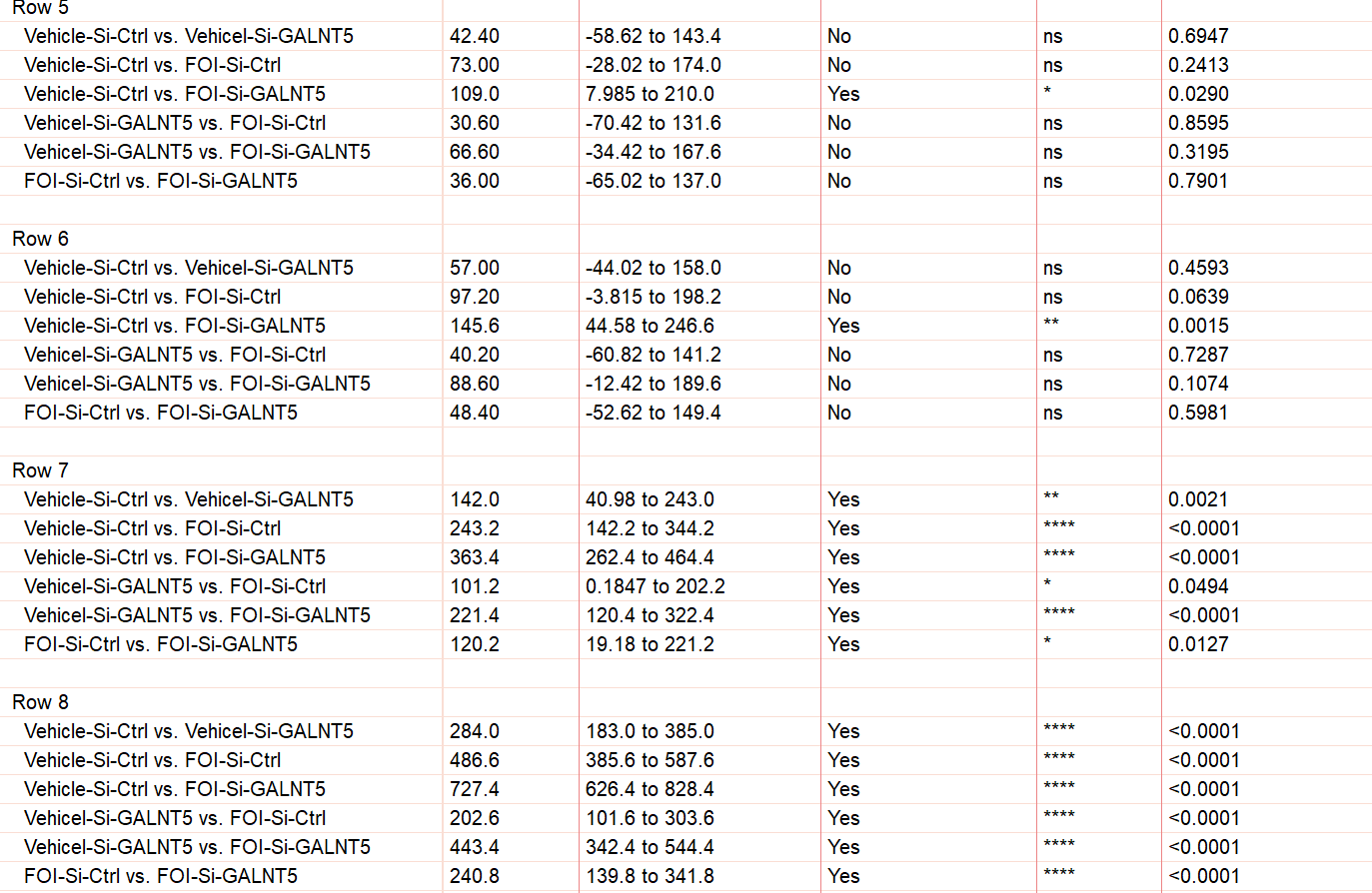

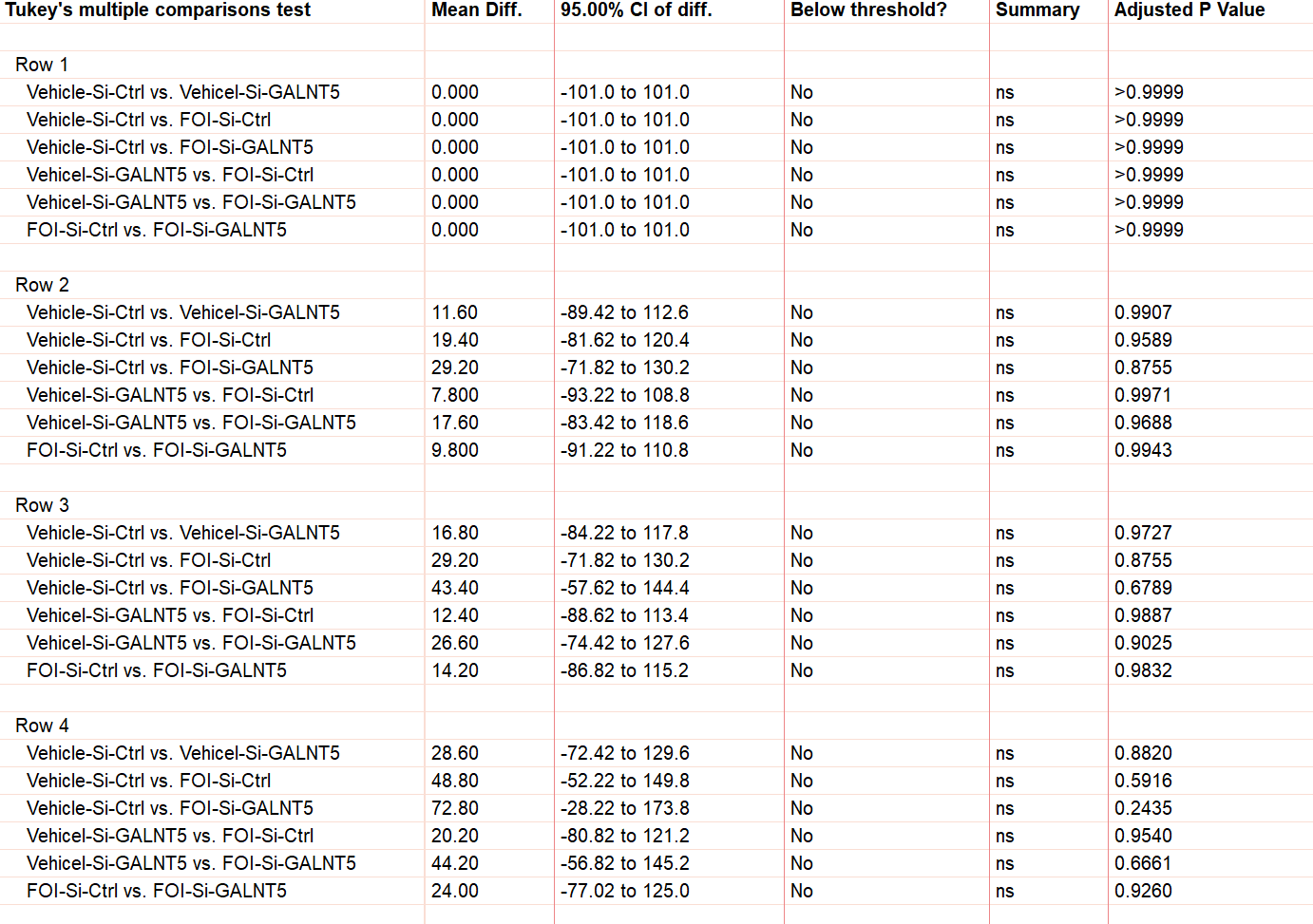
6H


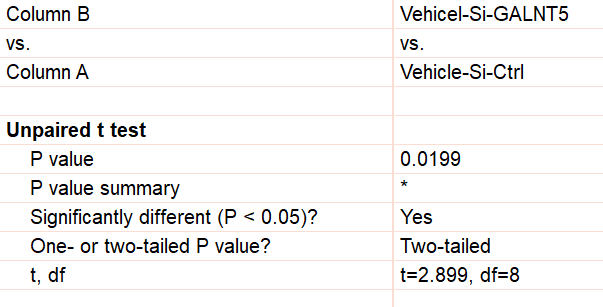
6I


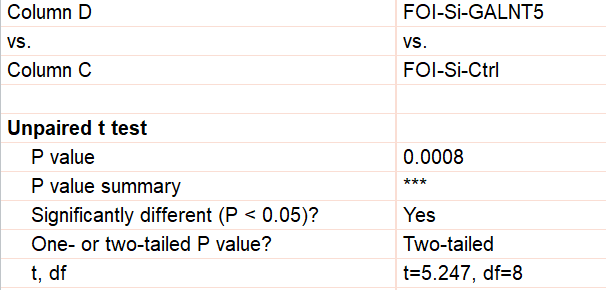

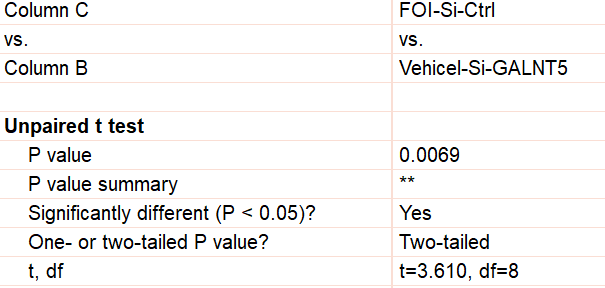


6J


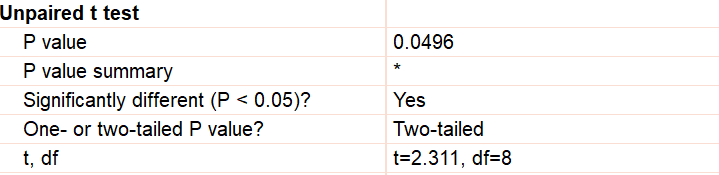


Figure7 p-value


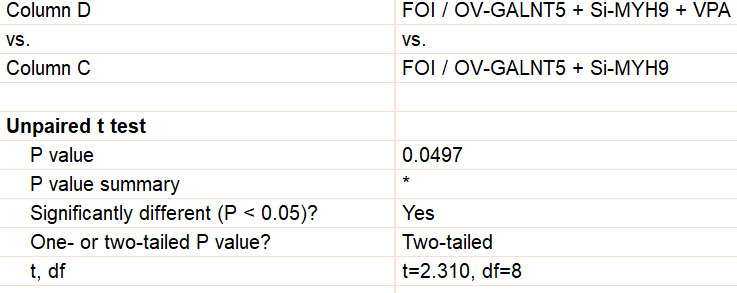

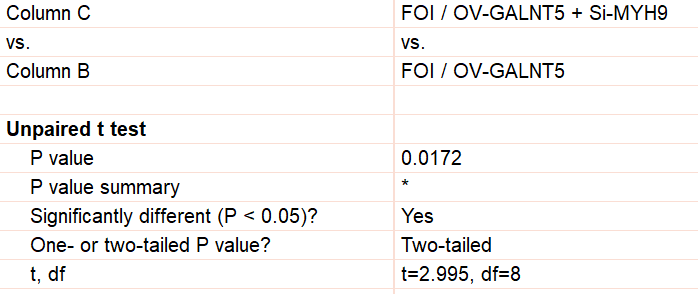

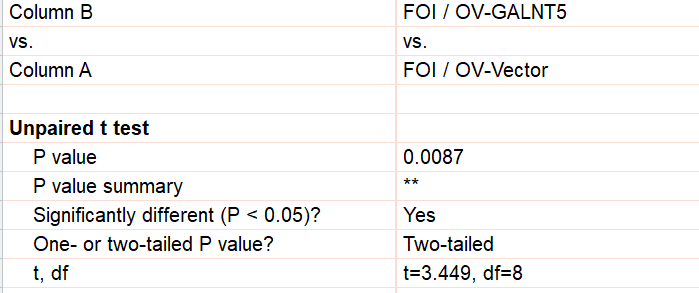
7C


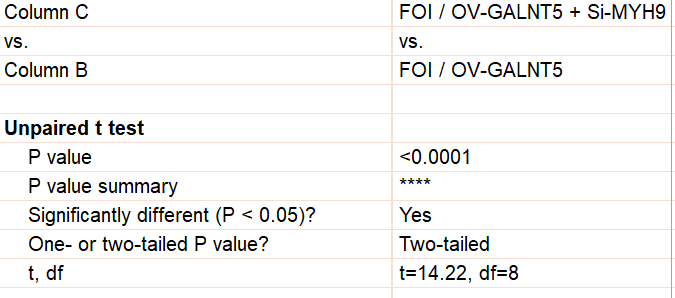

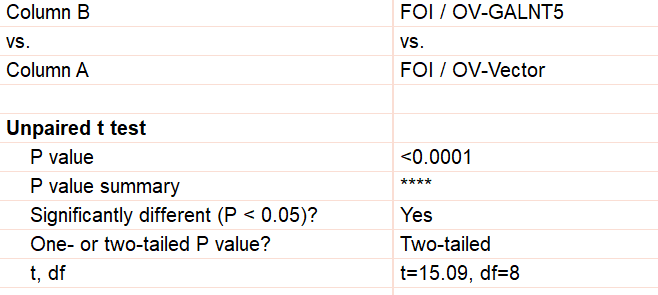
7D


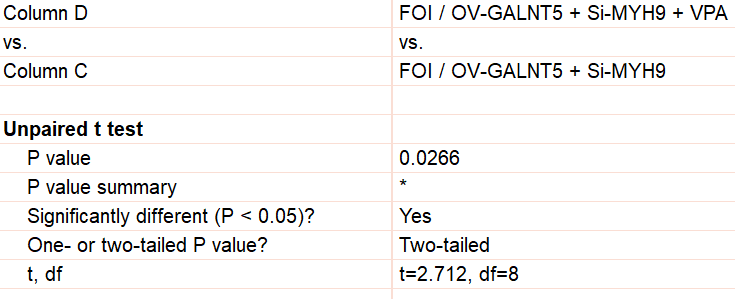


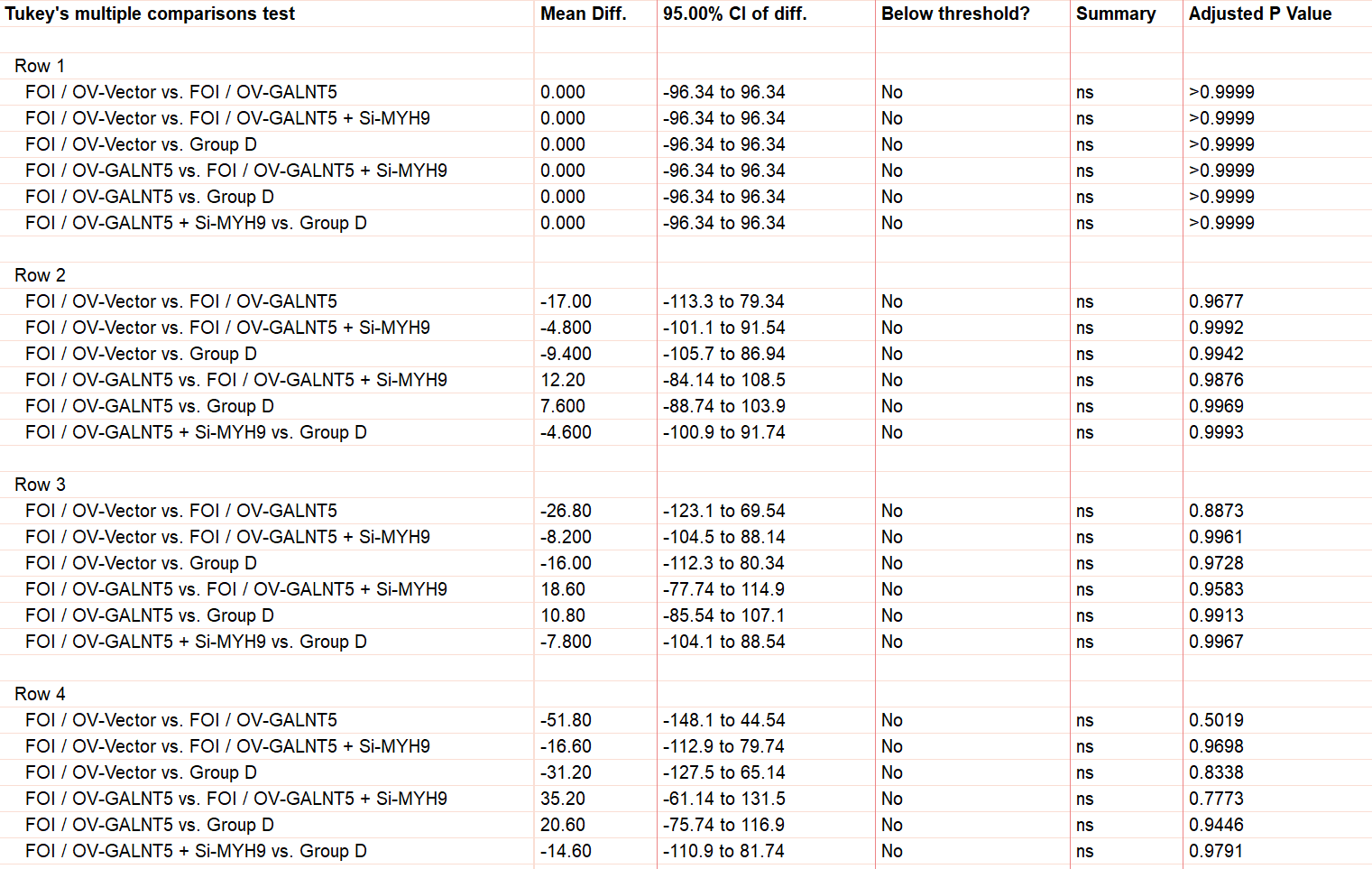
7F


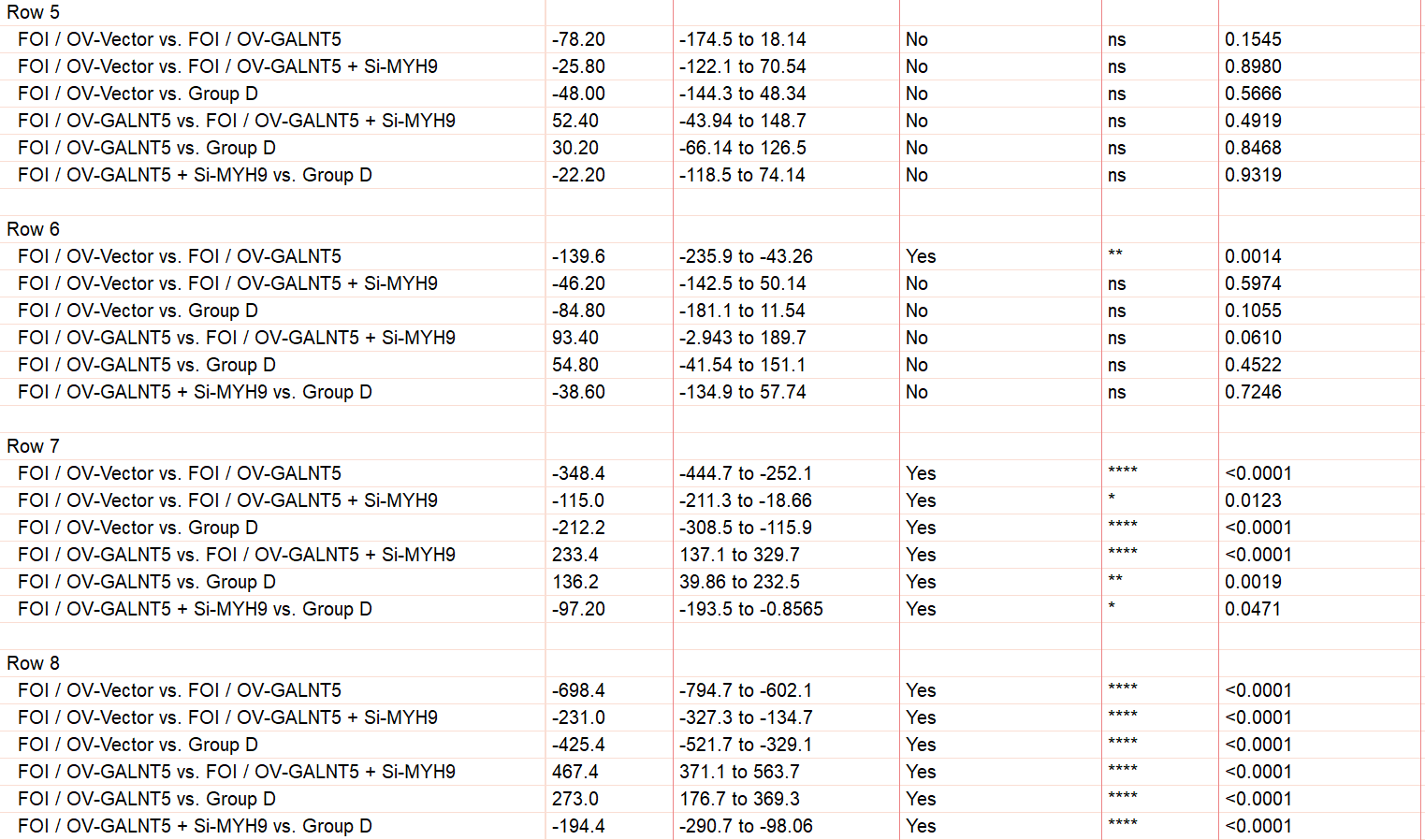


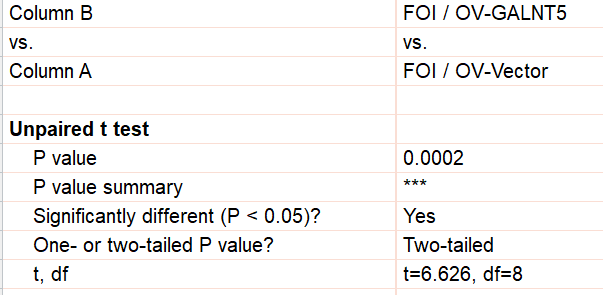
7G


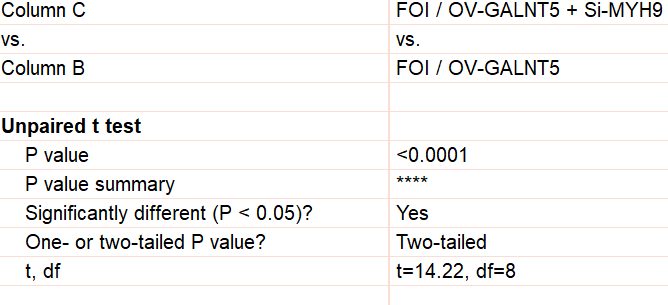


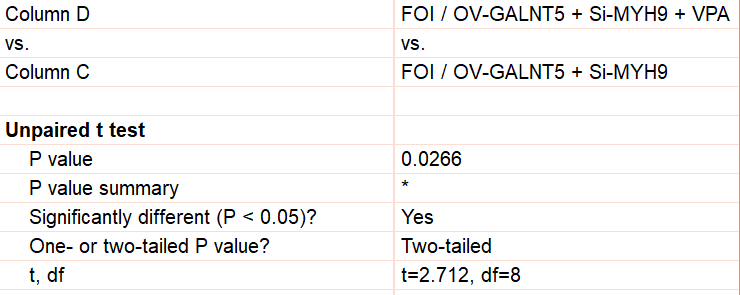

Supplement: Supplementary file 5 — P value [file 41419_2024_7110_MOESM5_ESM.docx]
